# Supplementary material for: Mitochondria‐associated membrane collapse is a common pathomechanism in SIGMAR1‐ and SOD1‐linked ALS
Source: EMBO Mol Med. 2016 Nov 7;8(12):1421–37. doi: 10.15252/emmm.201606403 (PMC5167132; doi:10.15252/emmm.201606403)
Supplement: Supplementary file 1 — Expanded View Figures PDF [file EMMM-8-1421-s001.pdf]

Expanded View Figures

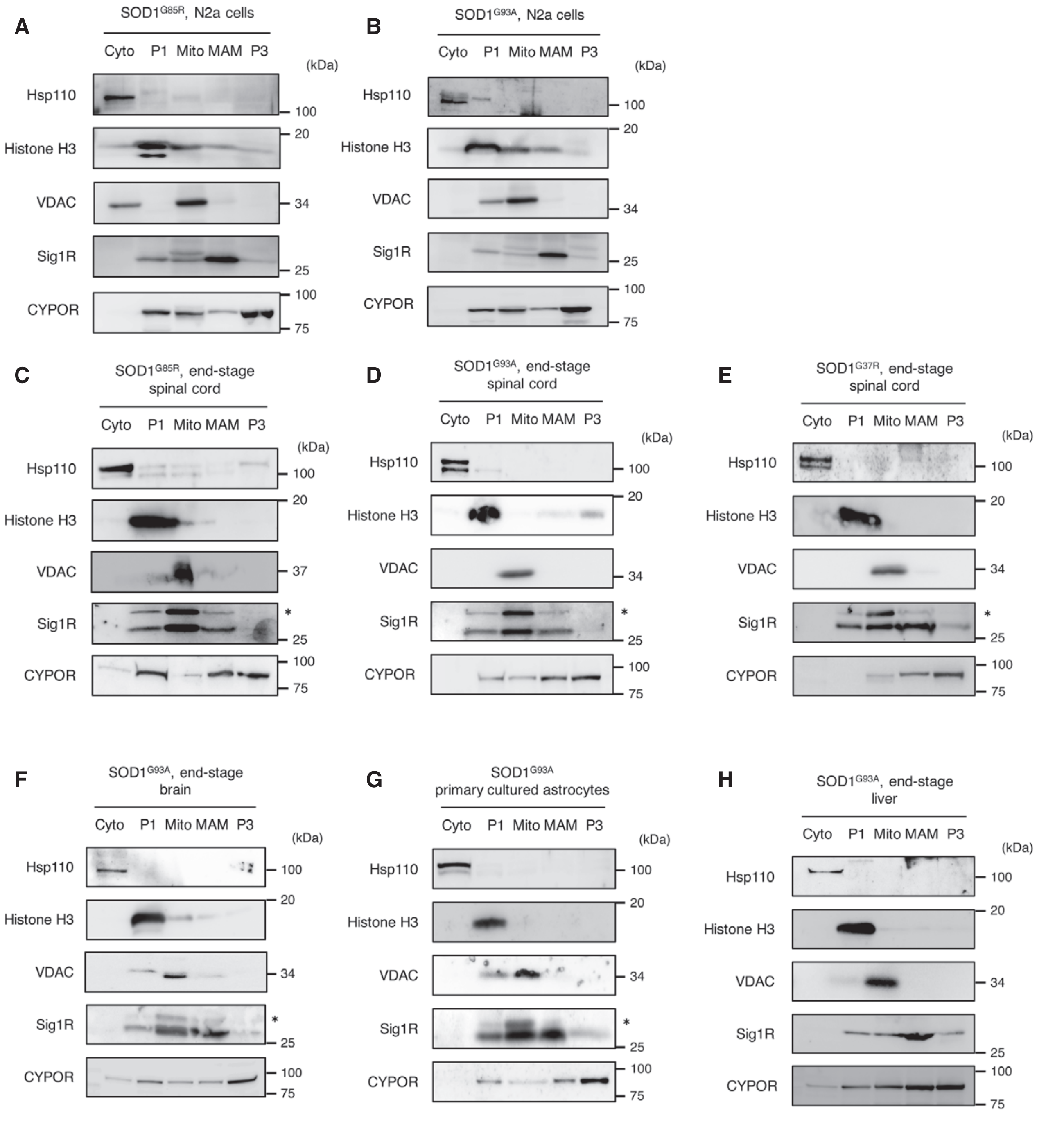

Figure EV1. Confirmation of the subcellular fractionation in various samples.

A–H Representative immunoblotting images of fractionated samples from N2a cells transfected with mutant SOD1 (A and B), spinal cords (C–E), brain (F), primary cultured astrocytes (G), or liver (H) from SOD1 transgenic mice. Representative blots from at least three independent experiments are shown. The asterisks indicate non-specific bands.

Source data are available online for this figure.

 © 2016 The Authors

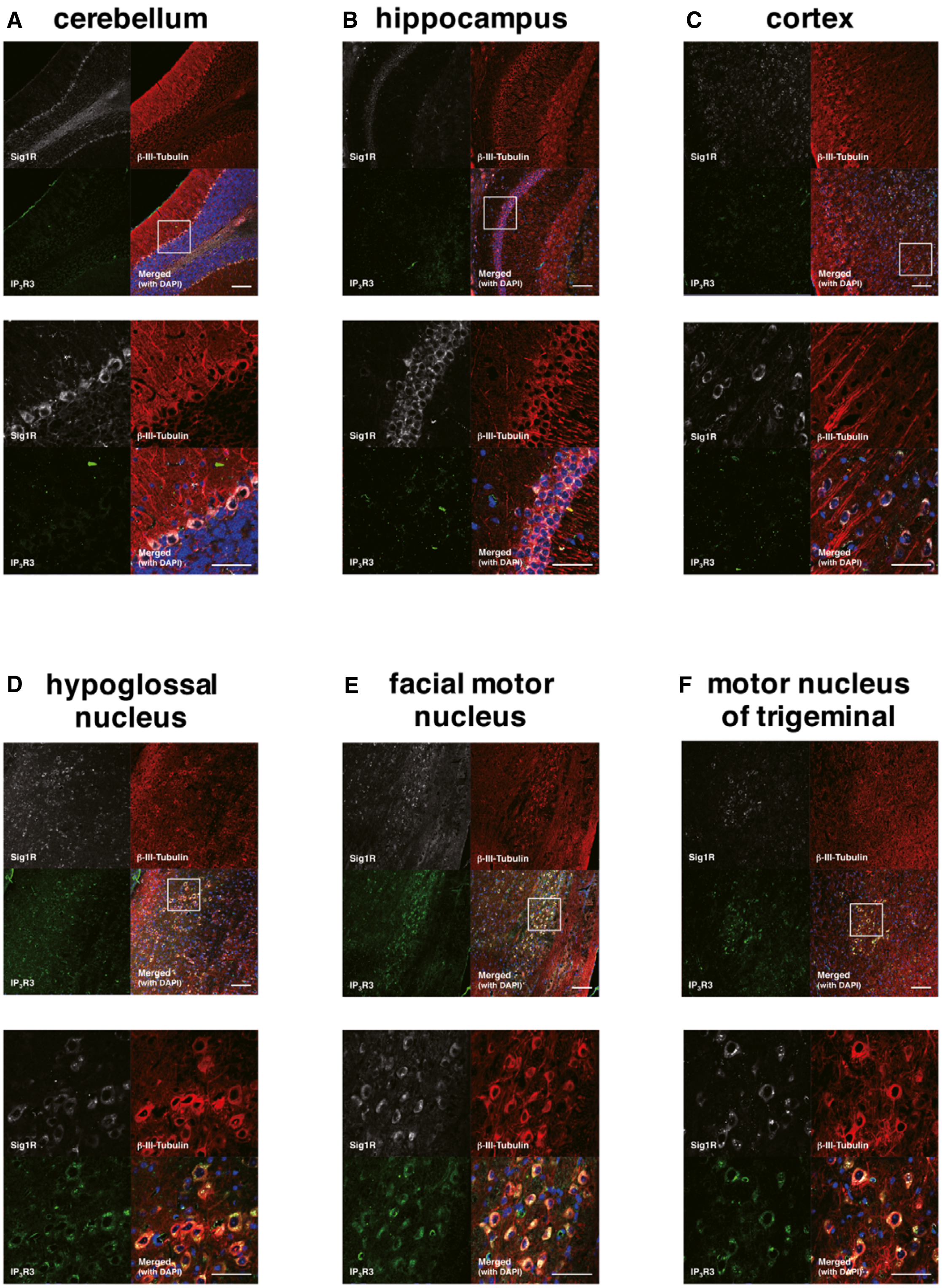

Figure EV2. Selective expression of IP_3R3 in the motor nuclei of mouse brain.

A–F Immunofluorescence staining of mouse brains from non-transgenic mice. Sagittal sections of mouse brains were stained using anti-Sig1R (white), βIII-tubulin (red), and IP_3R3 (green) antibodies. Sig1R was expressed in most of the neurons tested. However, IP_3R3 was not expressed in cerebellum (A), hippocampus (B), or cortex (C), but specifically co-localized with Sig1R in hypoglossal nucleus (D), facial motor nucleus (E), or motor nucleus of trigeminal (F), indicating selective expression in IP_3R3 in the motor-related nuclei. Scale bars: 50 μm. The white boxed areas were magnified in the images at bottom.

© 2016 The Authors

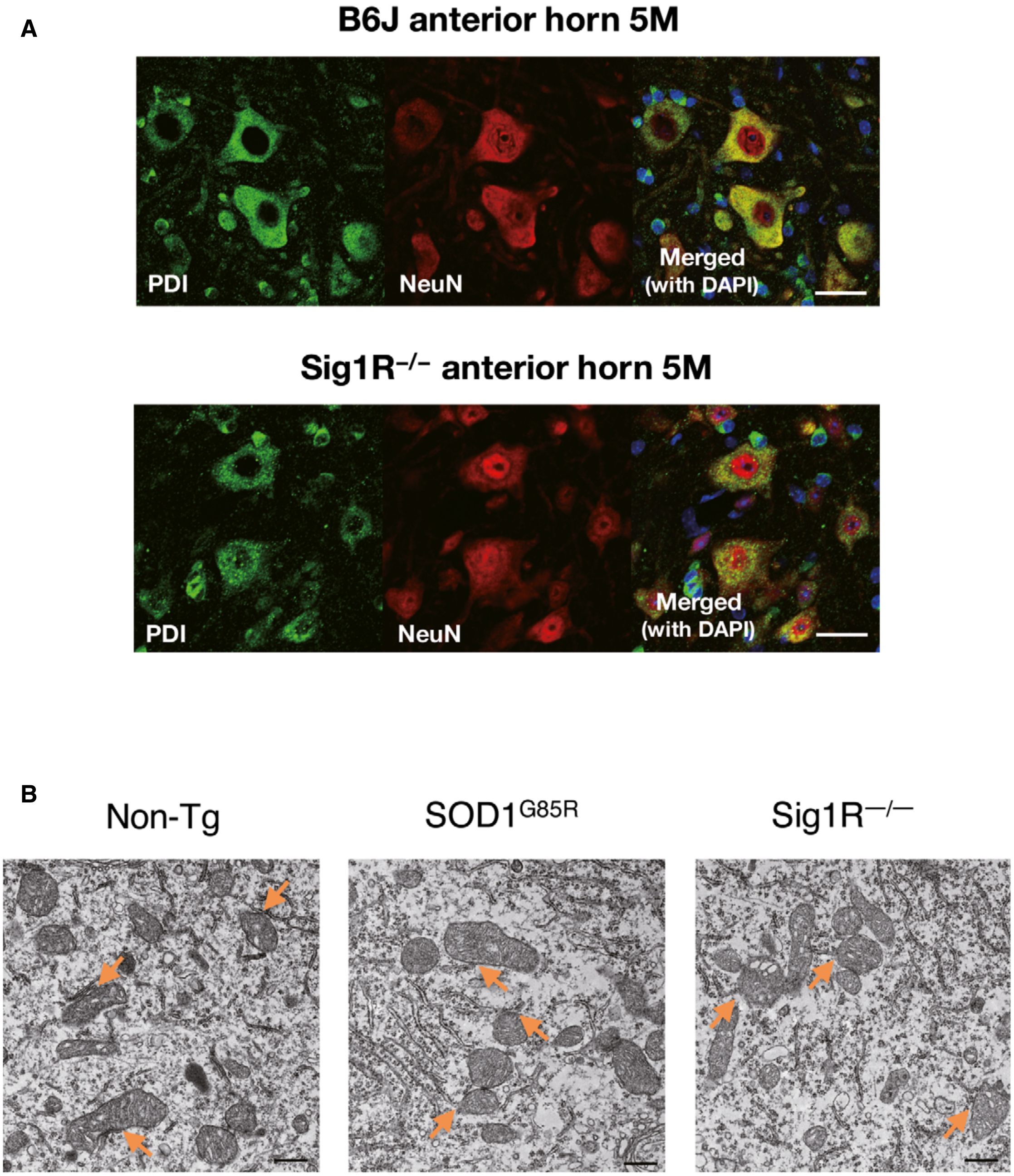

Figure EV3. Representative micrographs of ventral horn neurons in non-transgenic, $SOD1^{G85R}$, or $Sig1R^{-/-}$ mice.

A Immunofluorescence staining of mouse ventral horn neurons from non-transgenic or $Sig1R^{-/-}$ mice with anti-PDI and NeuN antibodies. Note that general ER morphology was not affected by the Sig1R deficiency. Scale bars: 50 μm.

B Representative low-magnification electron micrographs of the MAM (arrows) in motor neurons of 12-month-old non-transgenic (Non-Tg), $SOD1^{G85R}$, or $Sig1R^{-/-}$ mice. Scale bars: 500 nm.
