# Supplementary material for: Mitochondria‐associated membrane collapse is a common pathomechanism in SIGMAR1‐ and SOD1‐linked ALS
Source: EMBO Mol Med. 2016 Nov 7;8(12):1421–37. doi: 10.15252/emmm.201606403 (PMC5167132; doi:10.15252/emmm.201606403)
Supplement: Supplementary file 3 — Source Data for Expanded View [file EMMM-8-1421-s009.zip › EMM_06403_EV1_source_data/EMM_06403_EV1_source_data.pdf]

Fig. EV1A

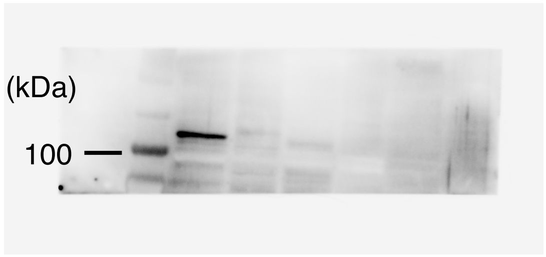

IB: Hsp110

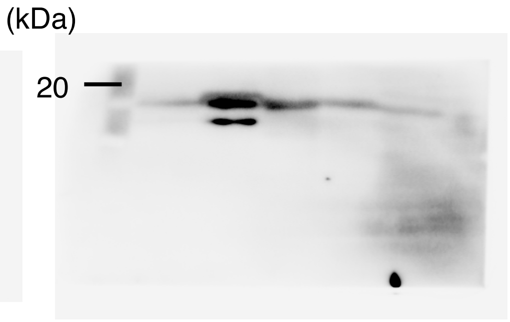

IB: Histone H3

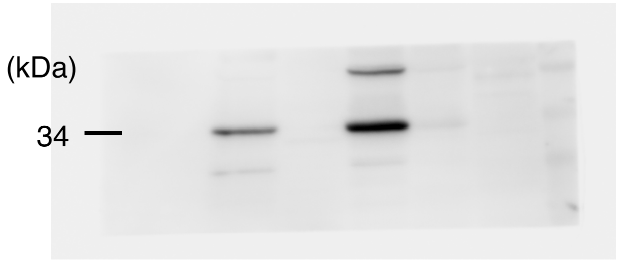

IB: VDAC

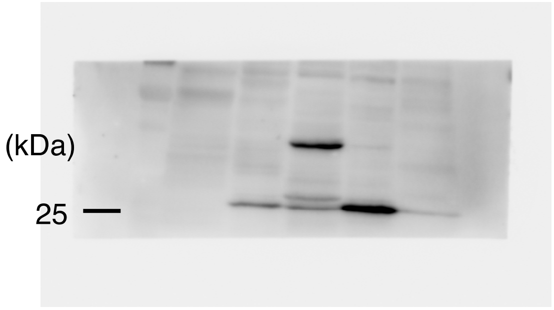

IB: Sig1R

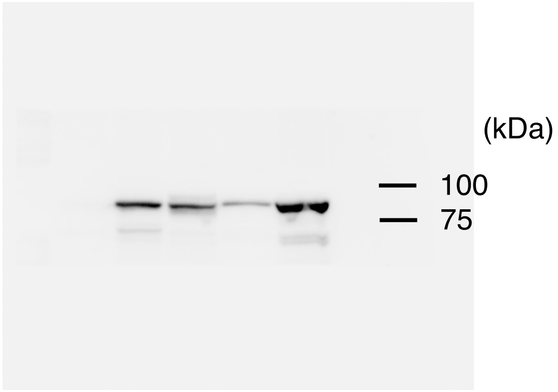

IB: CYPOR

Fig. EV1B

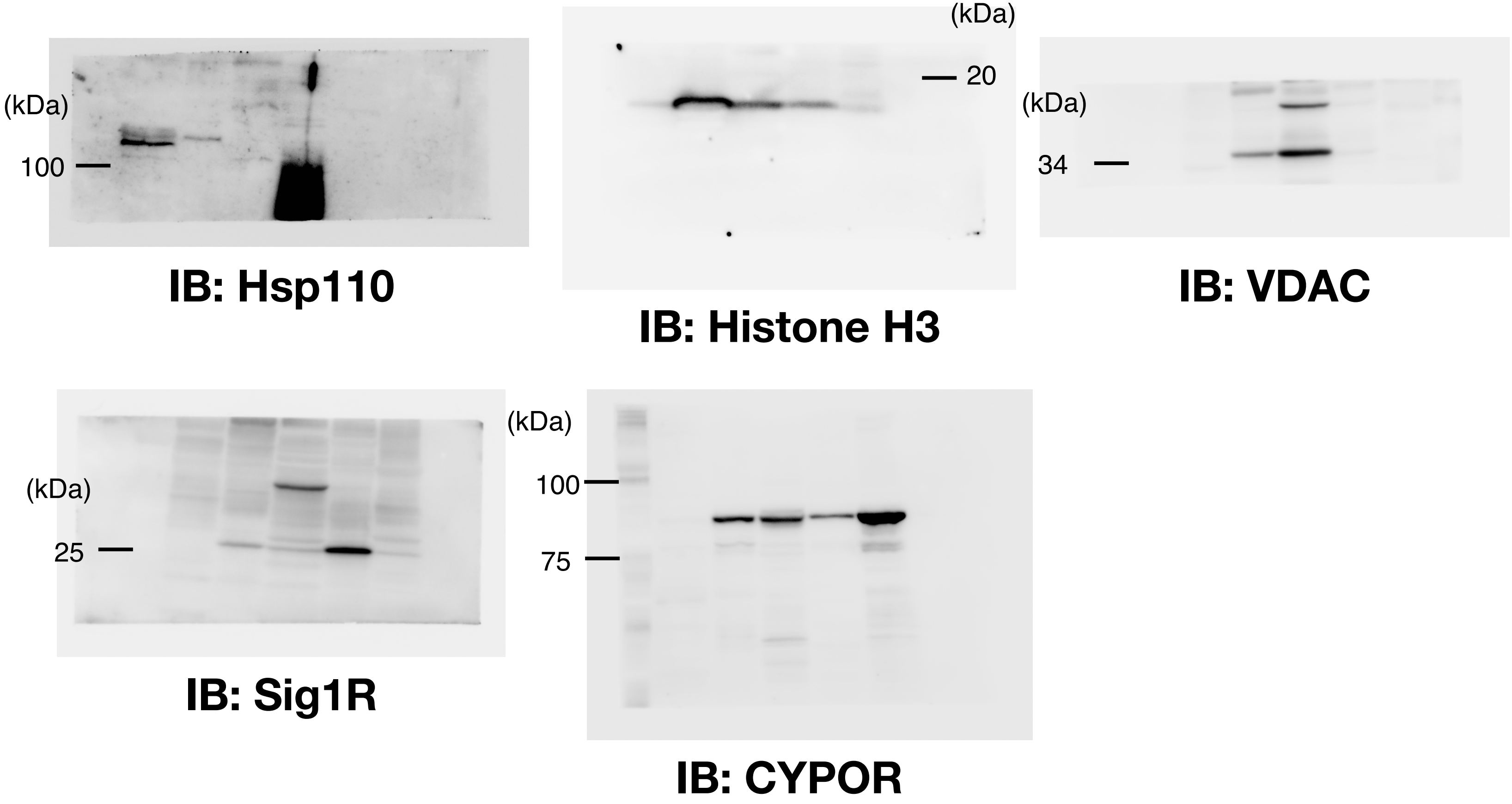
(kDa)
100
IB: Hsp110
(kDa)
20
IB: Histone H3
(kDa)
34
IB: VDAC
(kDa)
25
IB: Sig1R
(kDa)
100
75
IB: CYPOR

Fig. EV1C

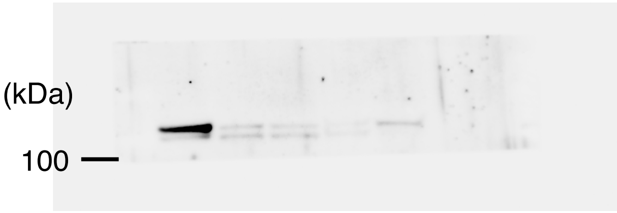

IB: Hsp110

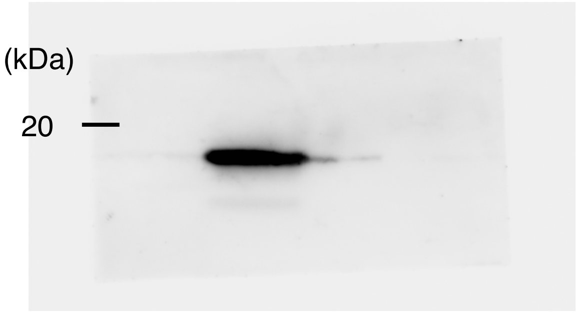

IB: Histone H3

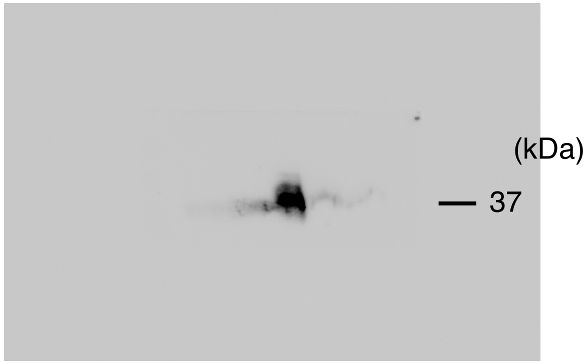

IB: VDAC

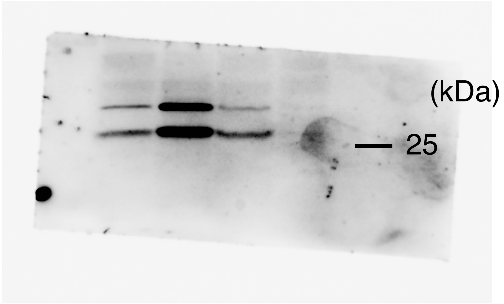

IB: Sig1R

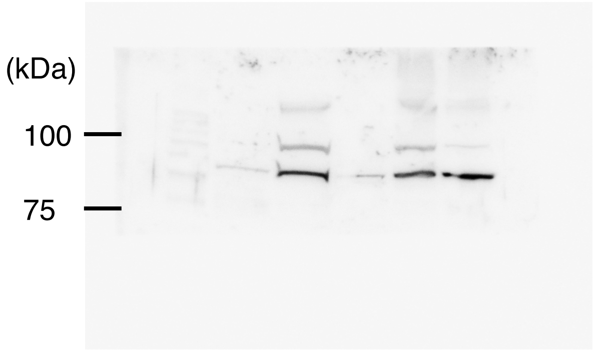

IB: CYPOR

Fig. EV1D

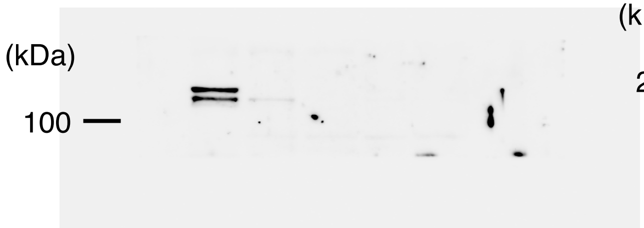

IB: Hsp110

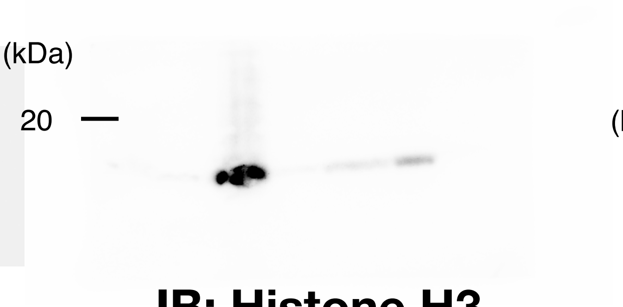

IB: Histone H3

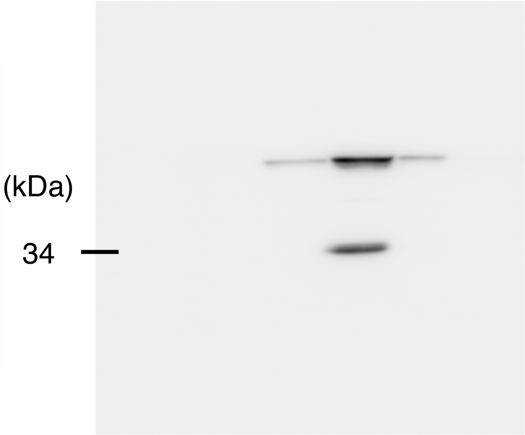

IB: VDAC

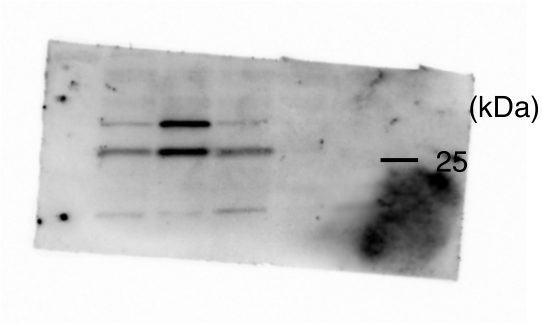

IB: Sig1R

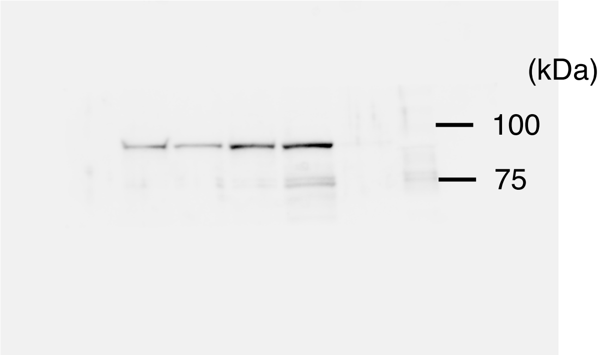

IB: CYPOR

Fig. EV1E

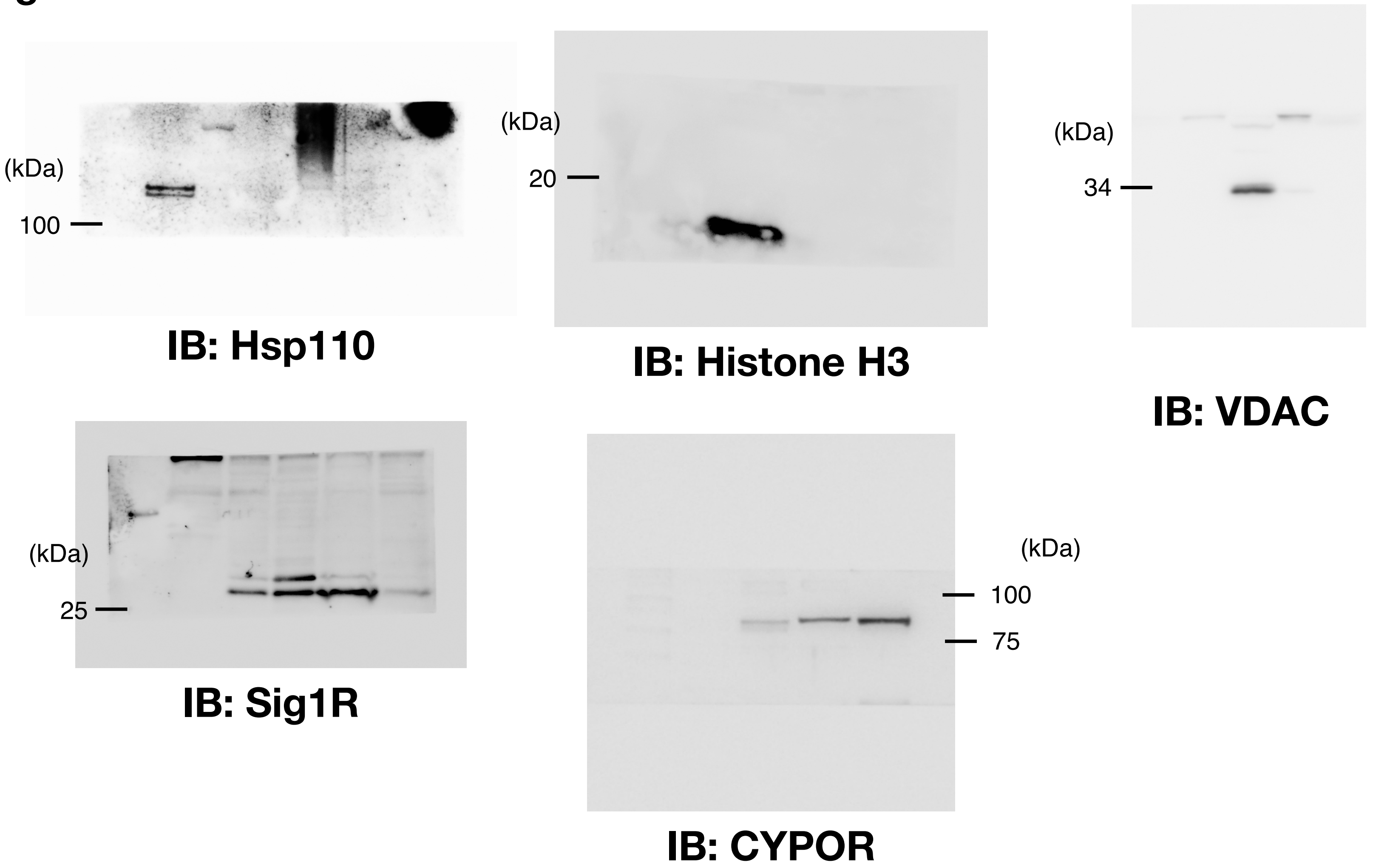
(kDa)
100
IB: Hsp110
(kDa)
20
IB: Histone H3
(kDa)
34
IB: VDAC
(kDa)
25
IB: Sig1R
(kDa)
100
75
IB: CYPOR

Fig. EV1F

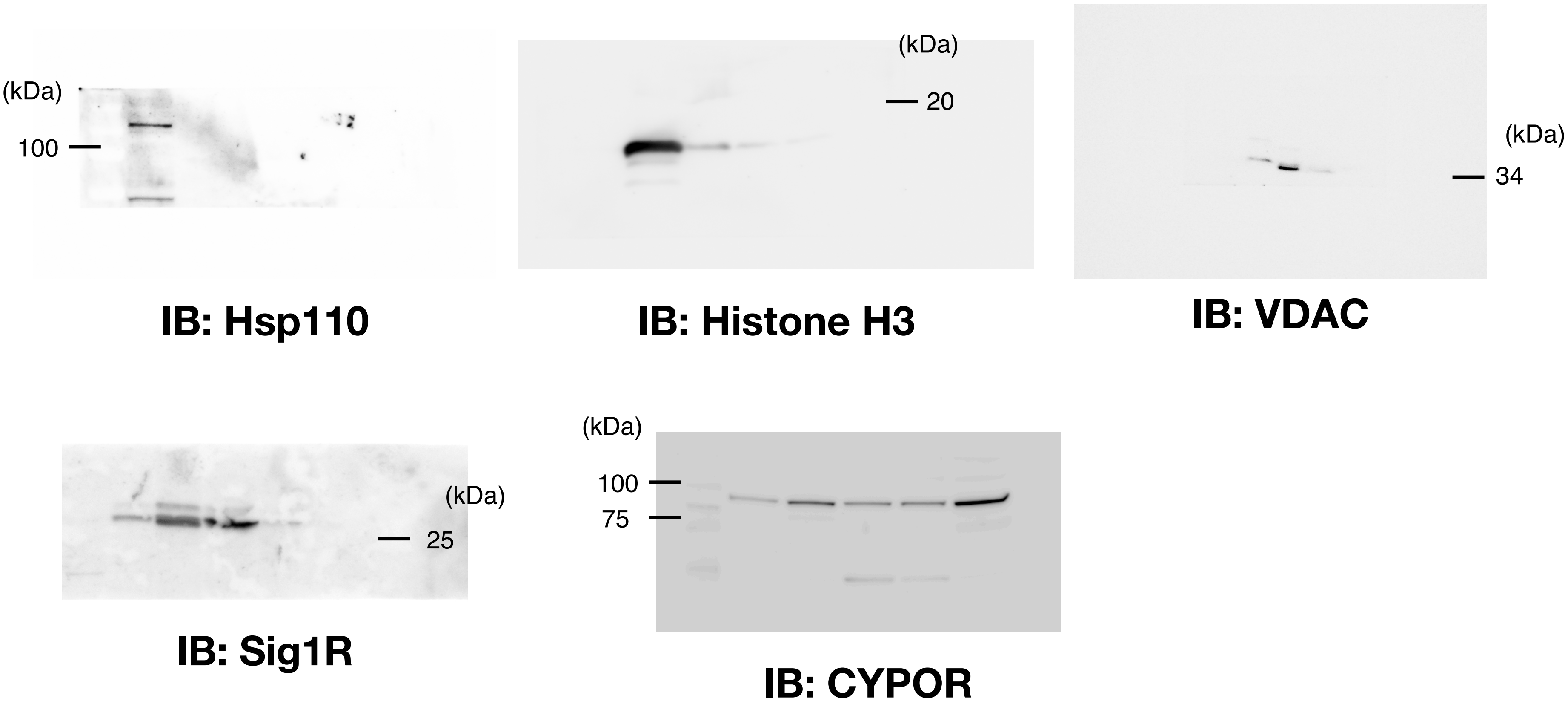
(kDa)
100
IB: Hsp110
(kDa)
20
IB: Histone H3
(kDa)
34
IB: VDAC
(kDa)
25
IB: Sig1R
(kDa)
100
75
IB: CYPOR

Fig. EV1G

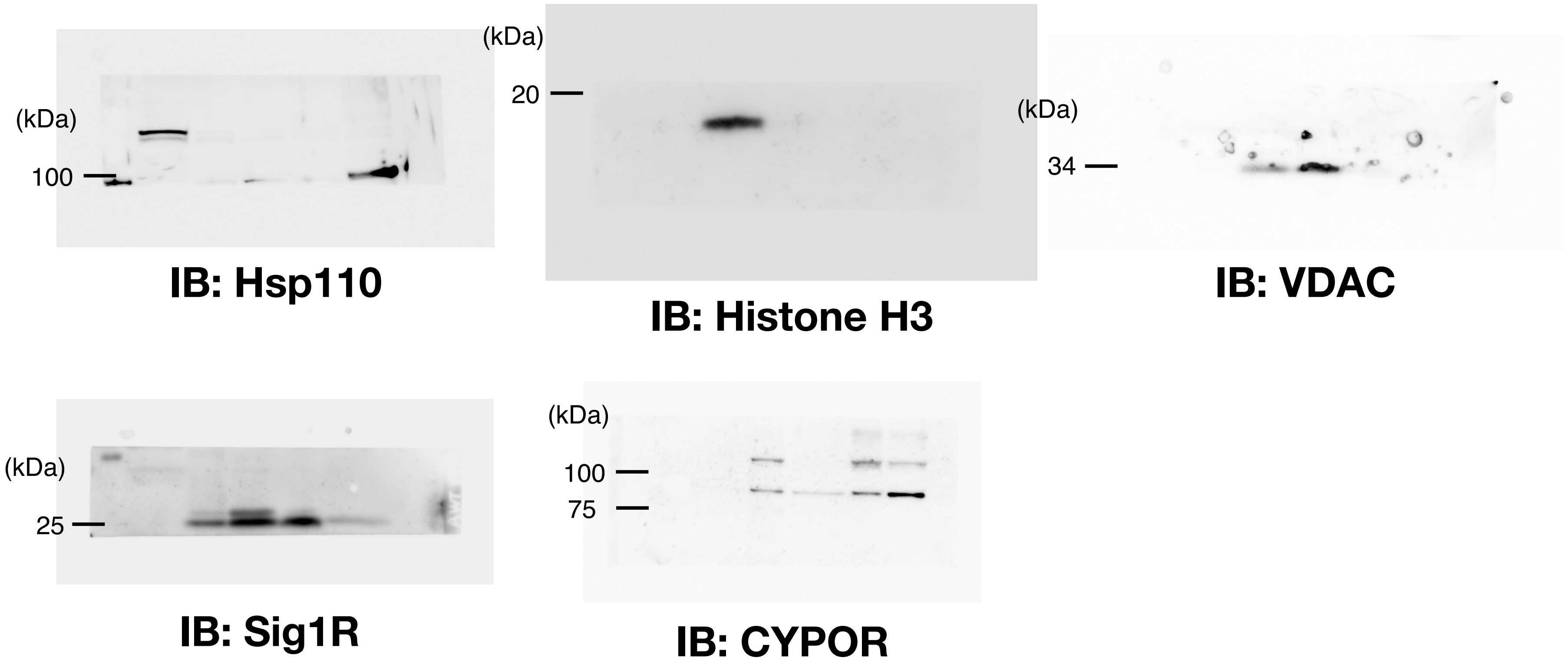

(kDa)
100
IB: Hsp110
(kDa)
20
IB: Histone H3
(kDa)
34
IB: VDAC
(kDa)
25
IB: Sig1R
(kDa)
100
75
IB: CYPOR

Fig. EV1H

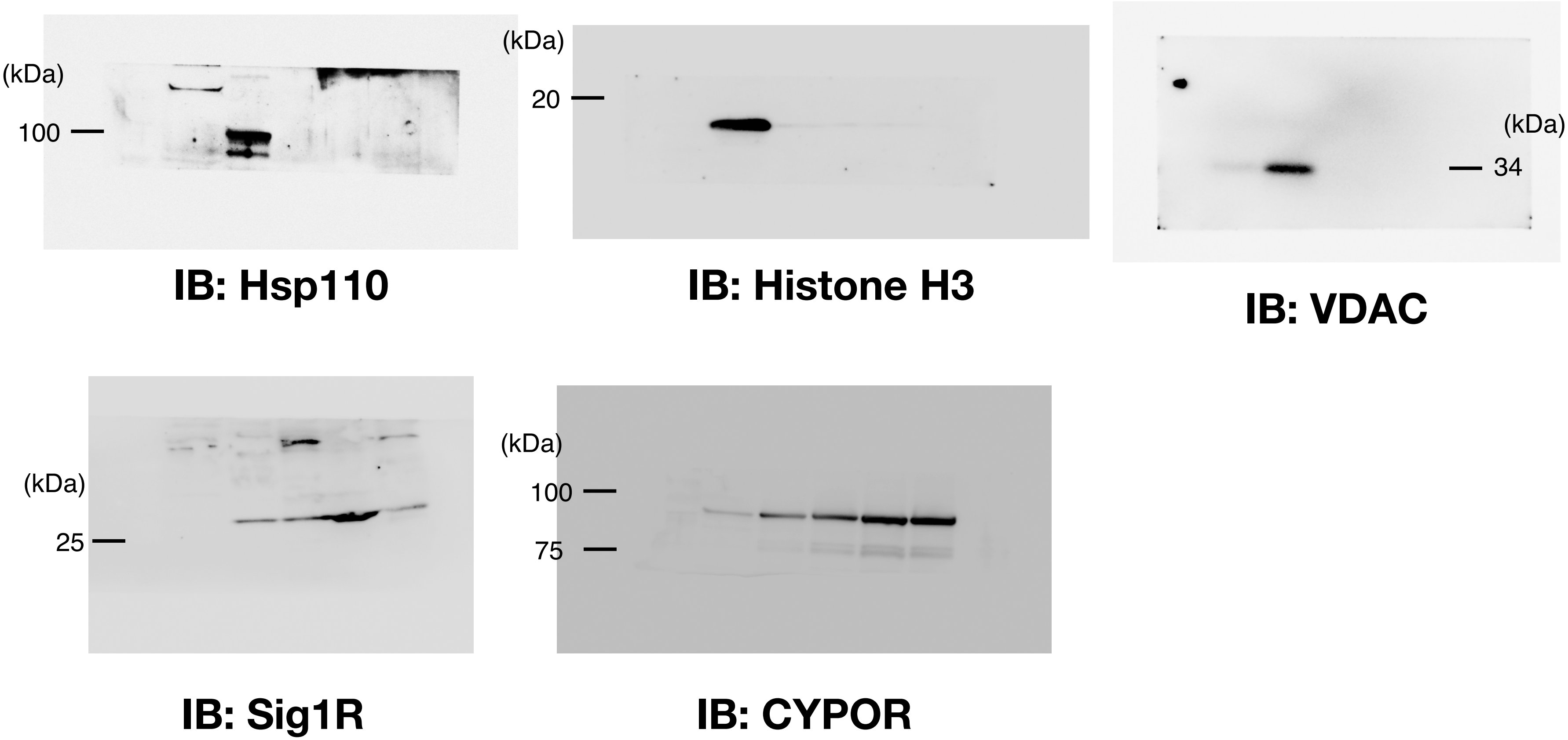
(kDa)
100
IB: Hsp110
(kDa)
20
IB: Histone H3
(kDa)
34
IB: VDAC
(kDa)
25
IB: Sig1R
(kDa)
100
75
IB: CYPOR
