# Supplementary material for: Mitochondria‐associated membrane collapse is a common pathomechanism in SIGMAR1‐ and SOD1‐linked ALS
Source: EMBO Mol Med. 2016 Nov 7;8(12):1421–37. doi: 10.15252/emmm.201606403 (PMC5167132; doi:10.15252/emmm.201606403)
Supplement: Supplementary file 5 — Source Data for Figure 2 [file EMMM-8-1421-s003.pdf]

Fig. 2A

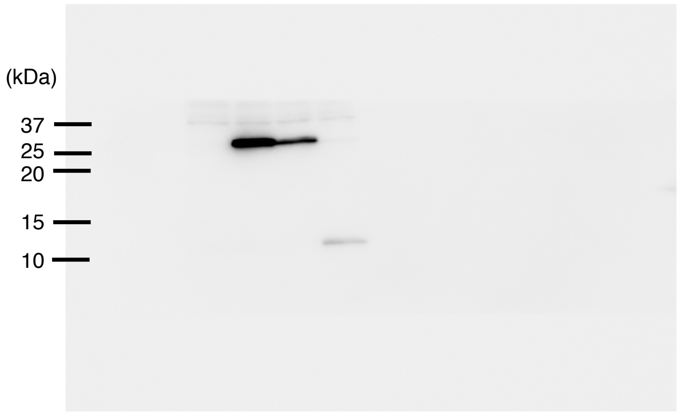

IB: FLAG(Sig1R)

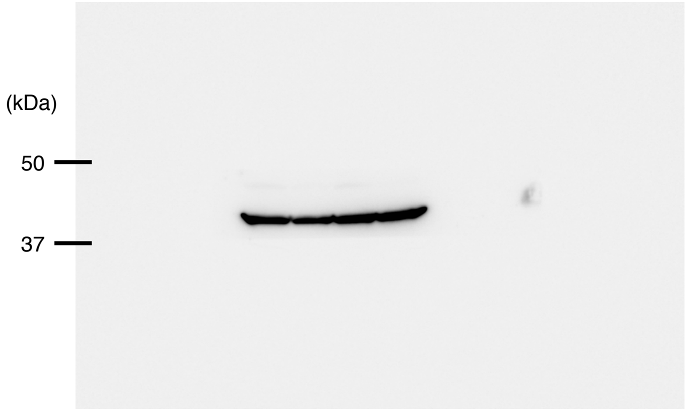

IB: actin

Fig. 2B

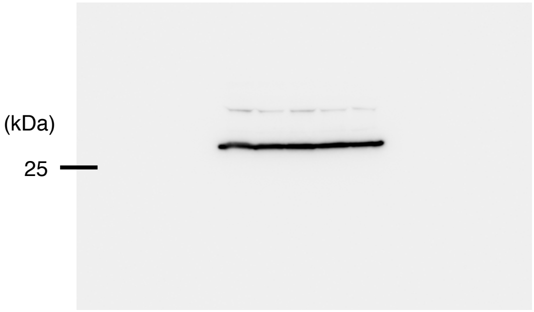

Wild type

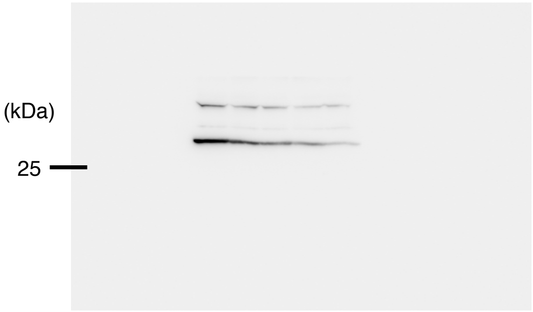

E102Q

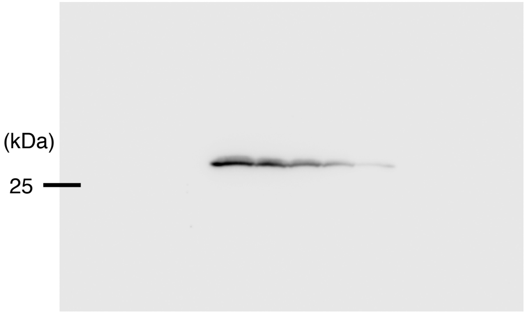

L95fs

IB: FLAG(Sig1R)

Fig. 2C

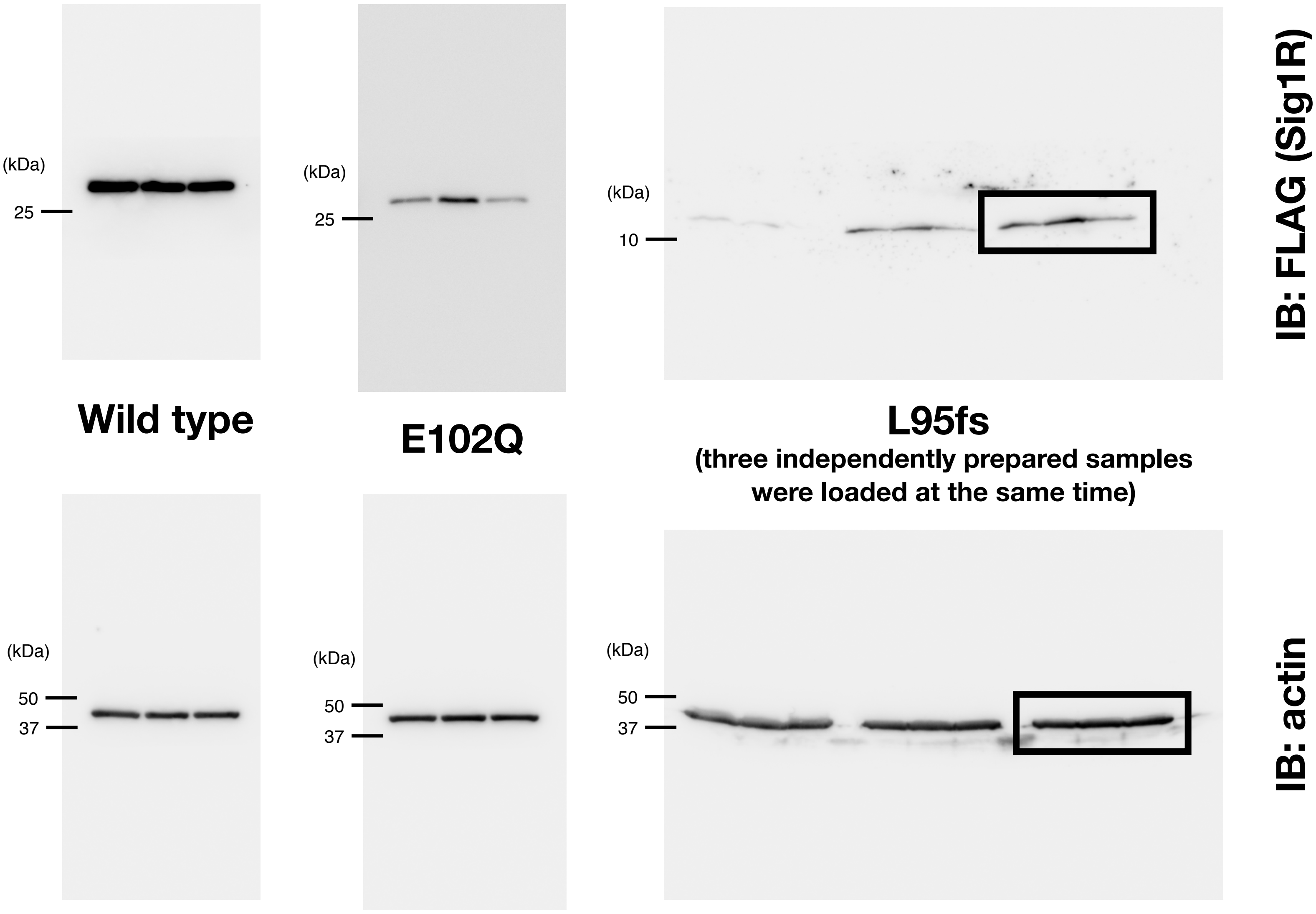
(kDa)
25
10
50
37
Wild type
E102Q
L95fs
(three independently prepared samples were loaded at the same time)
IB: FLAG (Sig1R)
IB: actin

Fig. 2E

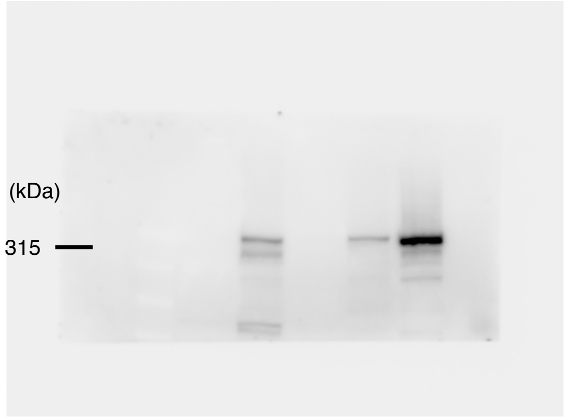

IB: IP_3R1

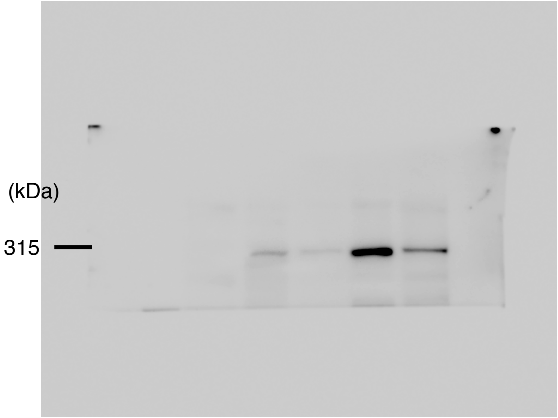

IB: IP_3R3

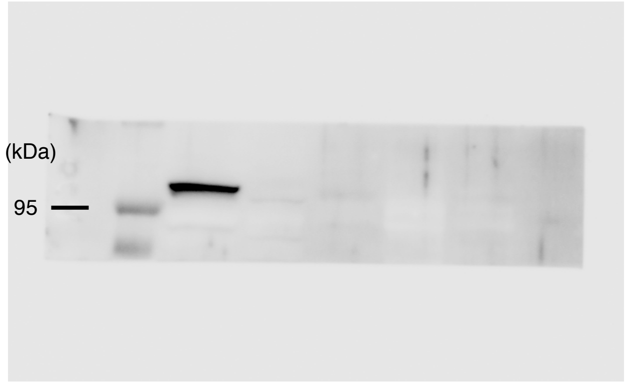

IB: Hsp110

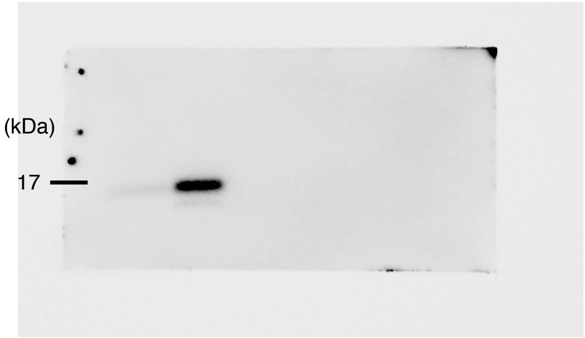

IB: Histone H3

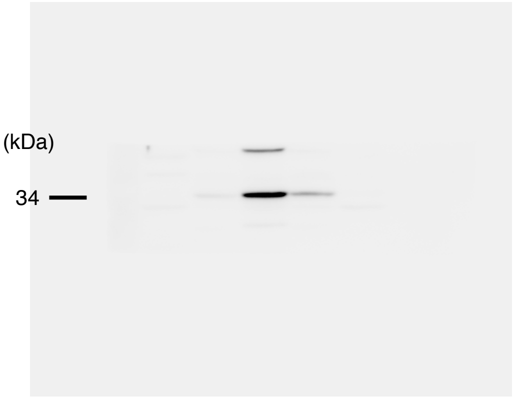

IB: VDAC

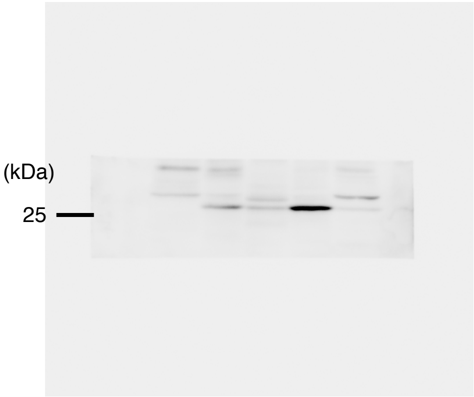

IB: Sig1R

Fig. 2E

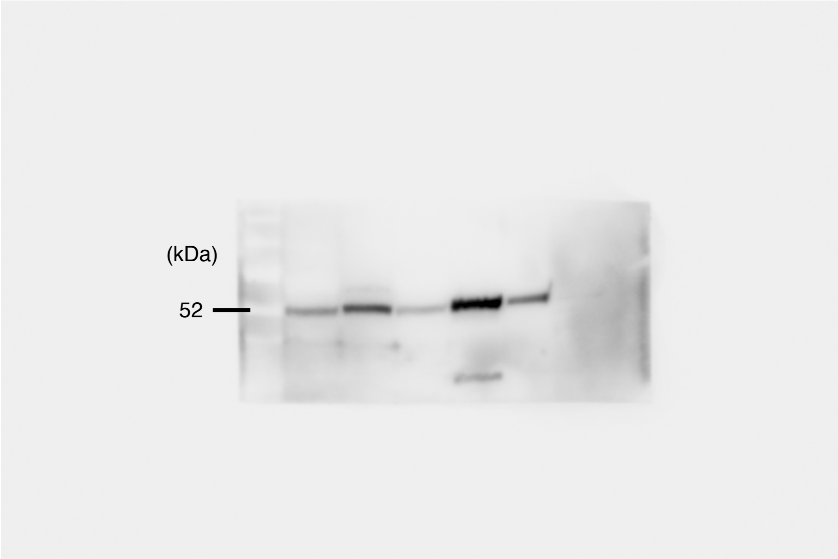

IB: Calreticulin

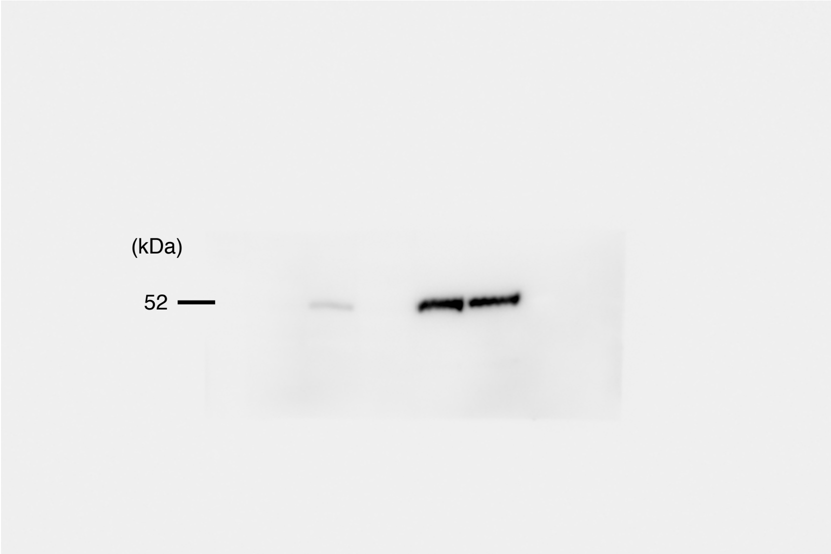

IB: PDI

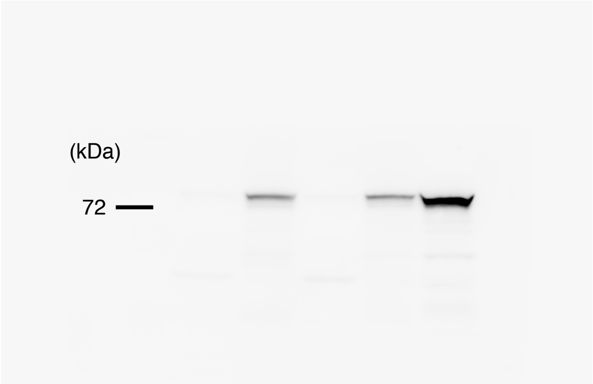

IB: CYPOR

Fig. 2F

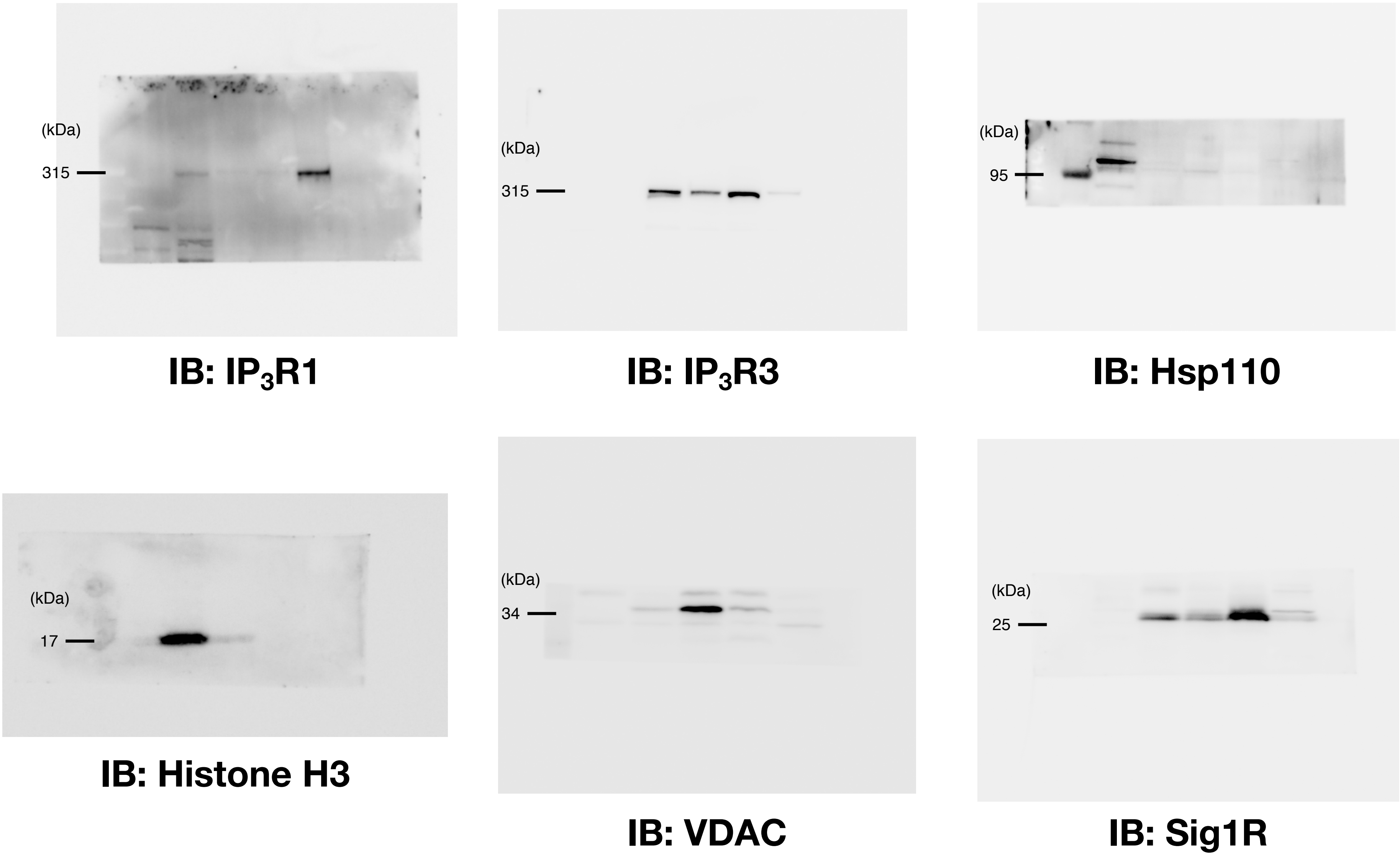

(kDa)
315
IB: IP$_3$R1
(kDa)
315
IB: IP$_3$R3
(kDa)
95
IB: Hsp110
(kDa)
17
IB: Histone H3
(kDa)
34
IB: VDAC
(kDa)
25
IB: Sig1R

Fig. 2F

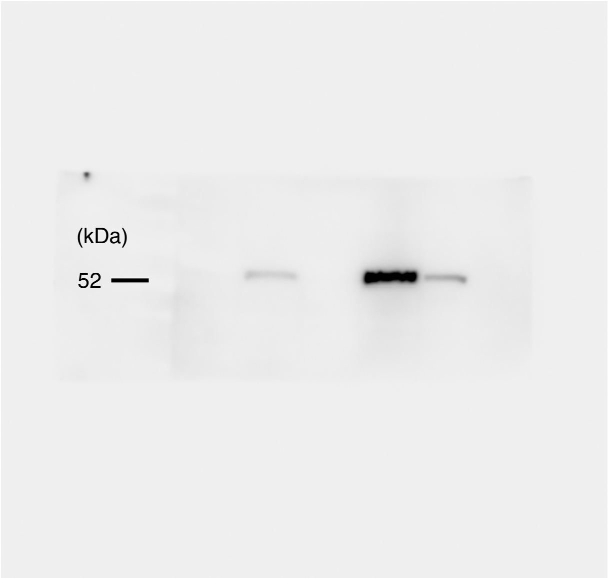

IB: Calreticulin

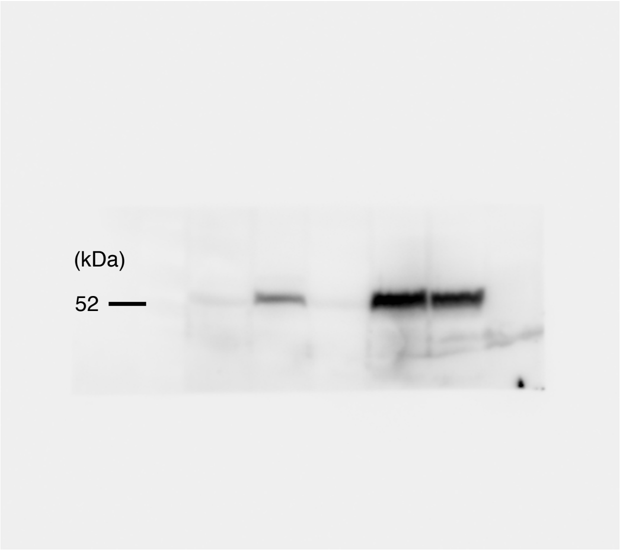

IB: PDI

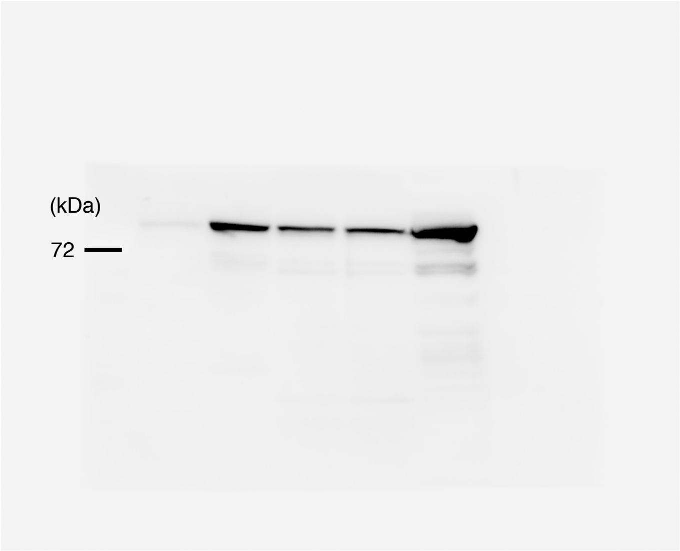

IB: CYPOR

Fig. 2G

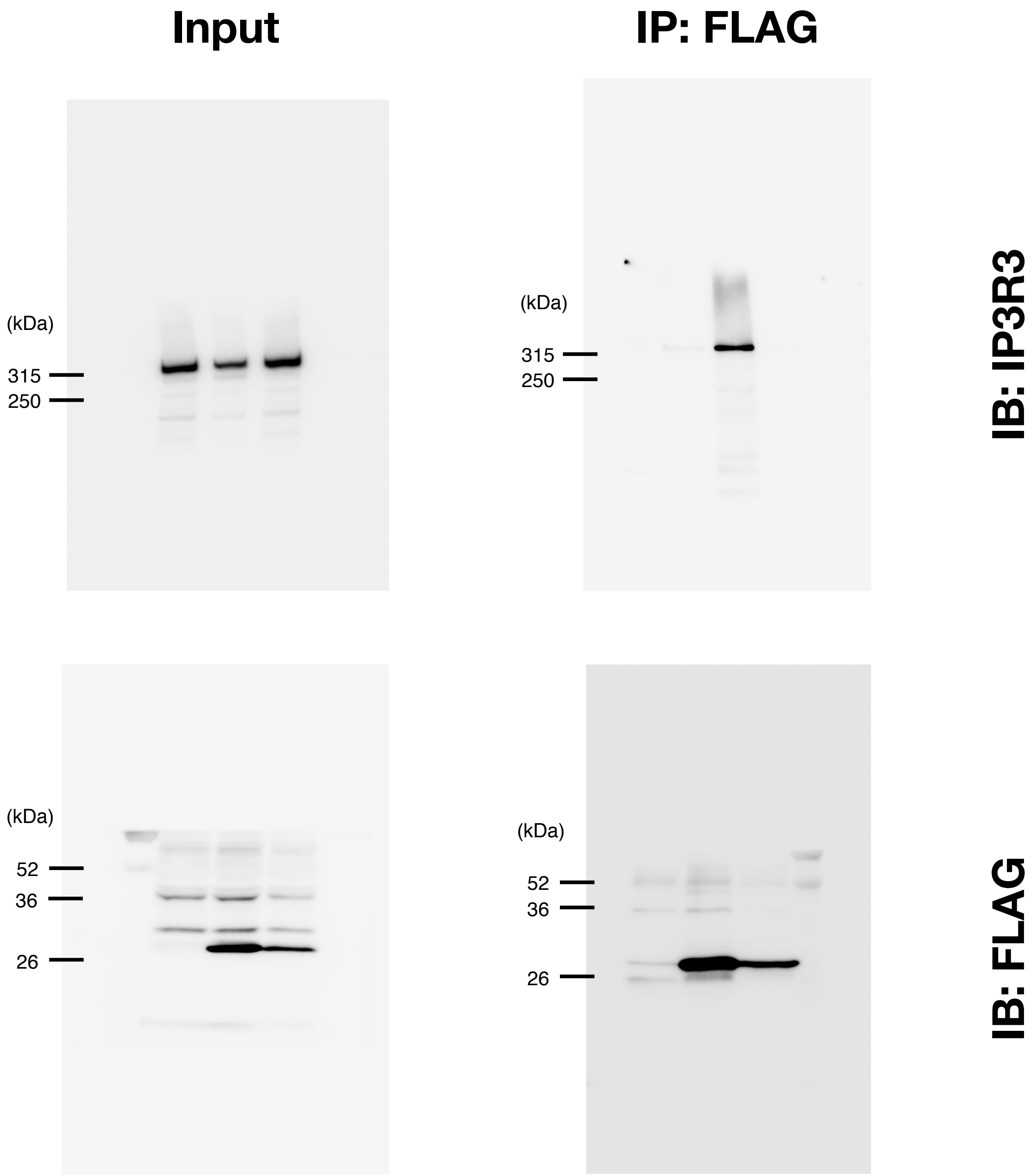
Input
IP: FLAG
(kDa)
315
250
(kDa)
315
250
IB: IP3R3
(kDa)
52
36
26
(kDa)
52
36
26
IB: FLAG

Fig. 2H

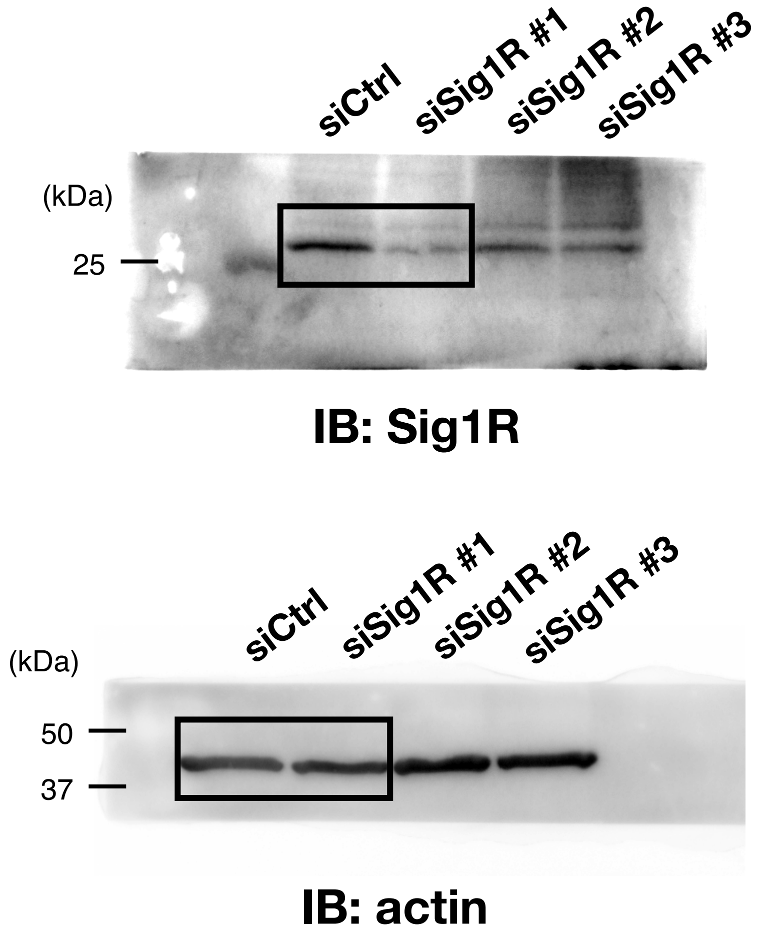

＊ siSig1R#1 is used in the other experiments in this study
