# Supplementary figures and images for: Mitochondria‐associated membrane collapse is a common pathomechanism in SIGMAR1‐ and SOD1‐linked ALS
Source: EMBO Mol Med. 2016 Nov 7;8(12):1421–37. doi: 10.15252/emmm.201606403 (PMC5167132; doi:10.15252/emmm.201606403)

**Fig. 3A**

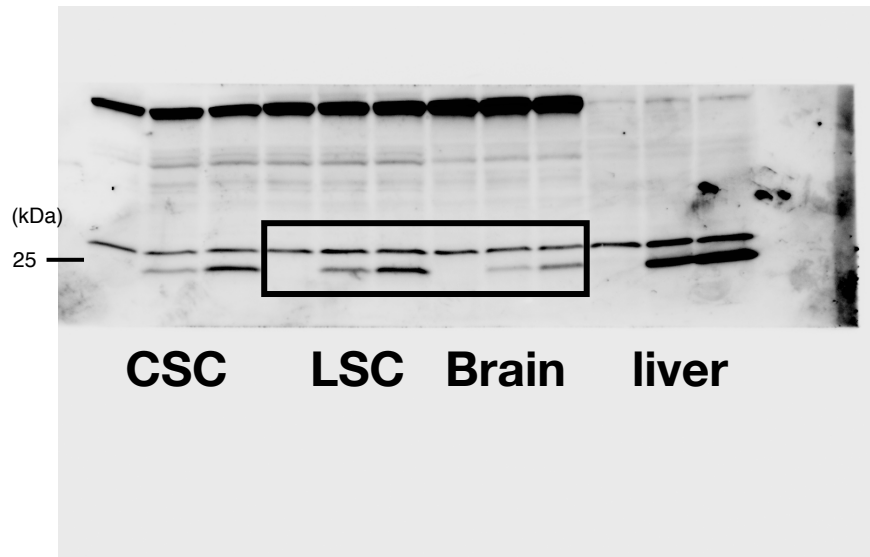

**IB: Sig1R**

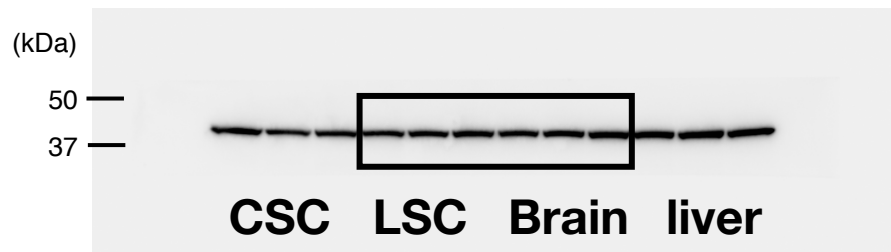

**IB: actin**

Supplement: Supplementary file 6 — Source Data for Figure 3 [file EMMM-8-1421-s004.pdf]

**Fig. 6L**

**IB: IP<sub>3</sub>R3**

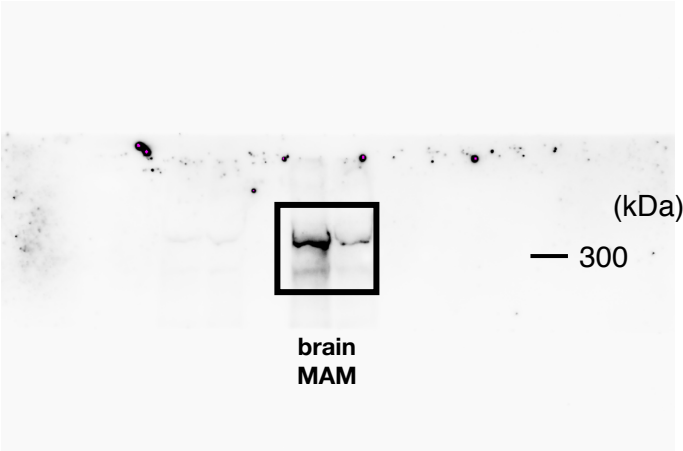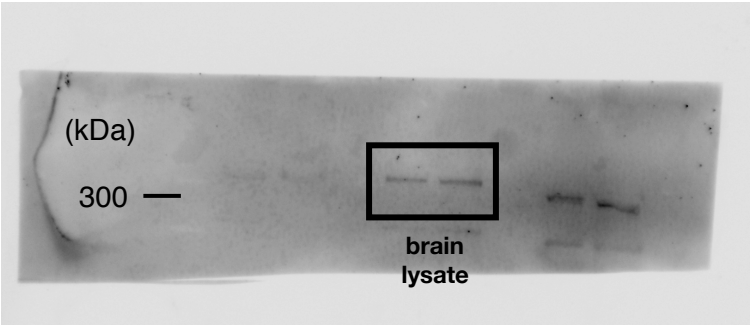

**IB: Calreticulin**

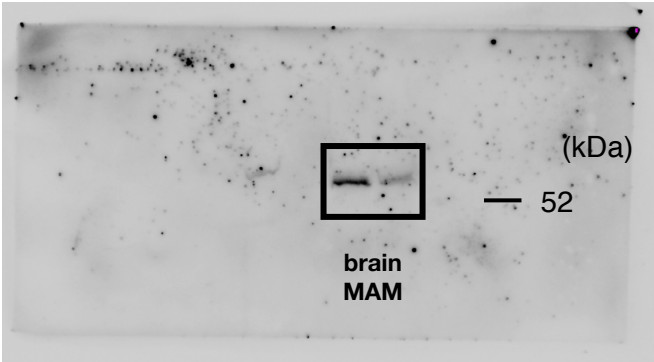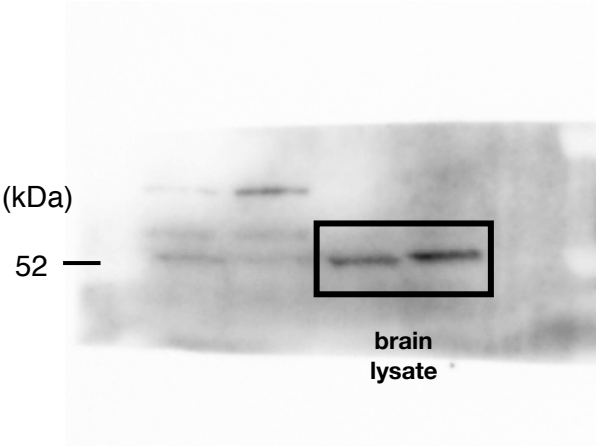

**IB: Sig1R**

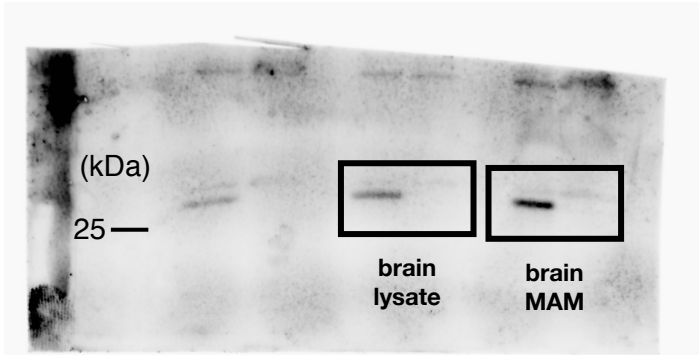

Supplement: Supplementary file 9 — Source Data for Figure 6 [file EMMM-8-1421-s007.pdf]

**Fig. 7H**

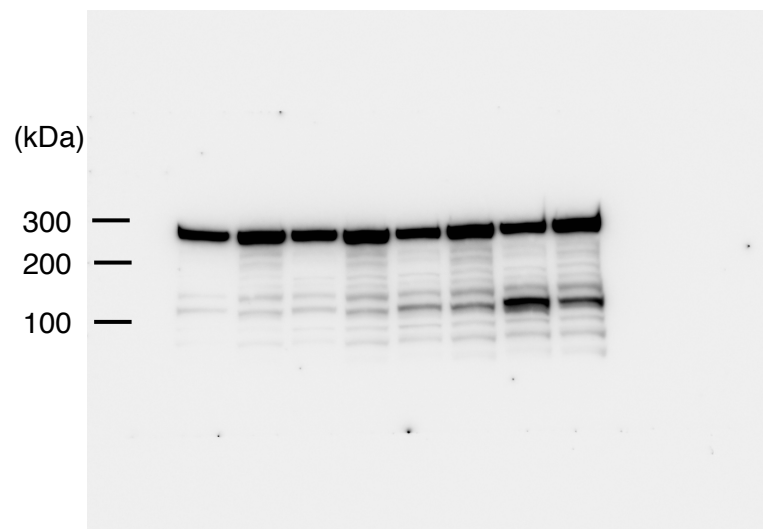

**IB: Spectrin  $\alpha$  II**

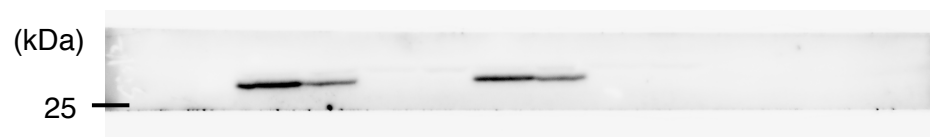

**IB: Sig1R**

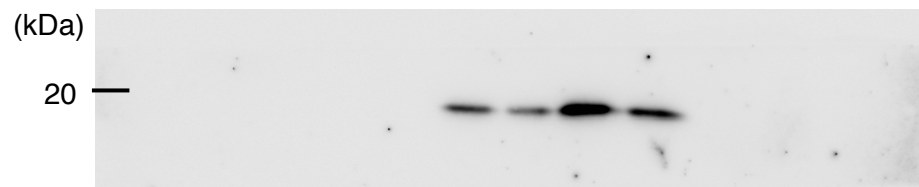

**IB: SOD1**

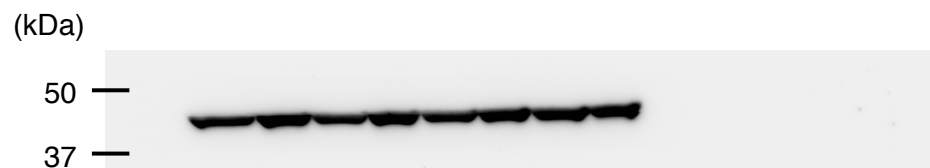

**IB: actin**

Supplement: Supplementary file 10 — Source Data for Figure 7 [file EMMM-8-1421-s008.pdf]
