# Supplementary material for: Mitochondria‐associated membrane collapse is a common pathomechanism in SIGMAR1‐ and SOD1‐linked ALS
Source: EMBO Mol Med. 2016 Nov 7;8(12):1421–37. doi: 10.15252/emmm.201606403 (PMC5167132; doi:10.15252/emmm.201606403)
Supplement: Supplementary file 7 — Source Data for Figure 4 [file EMMM-8-1421-s005.pdf]

Fig. 4A

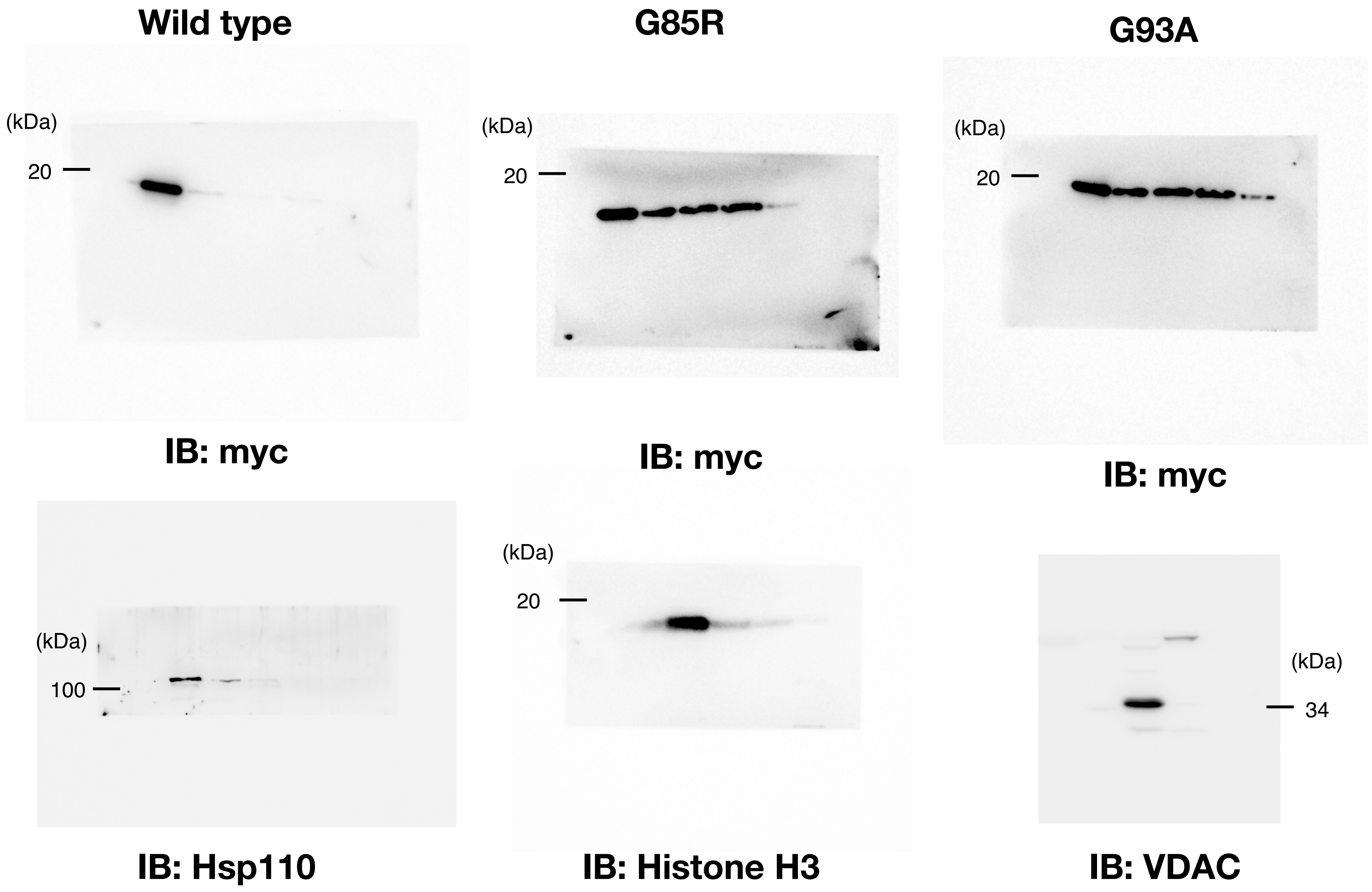
Wild type
G85R
G93A
(kDa)
20
IB: myc
IB: myc
IB: myc
(kDa)
100
IB: Hsp110
(kDa)
20
IB: Histone H3
(kDa)
34
IB: VDAC

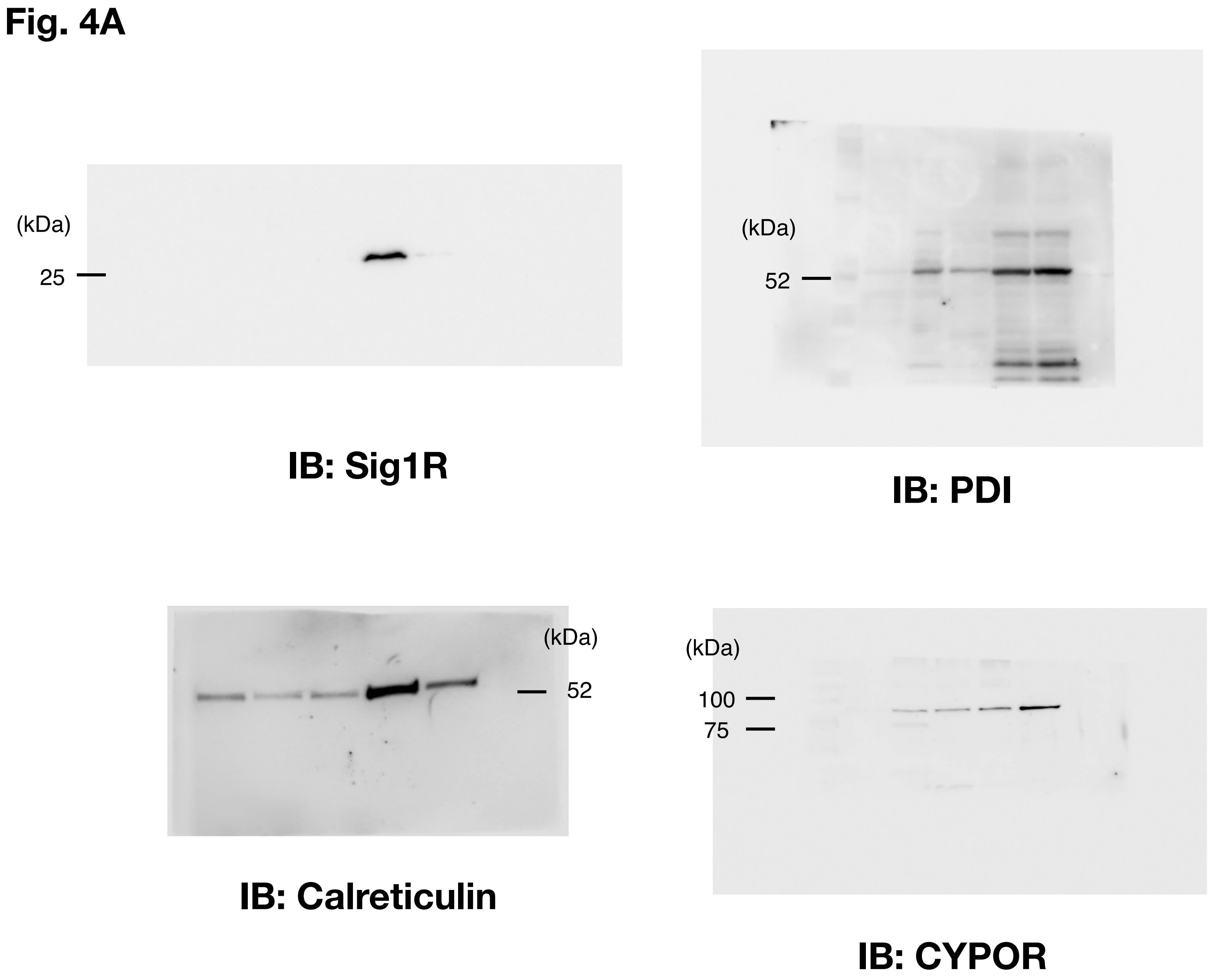
Fig. 4A
(kDa)
25
IB: Sig1R
(kDa)
52
IB: PDI
(kDa)
52
IB: Calreticulin
(kDa)
100
75
IB: CYPOR

Fig. 4B

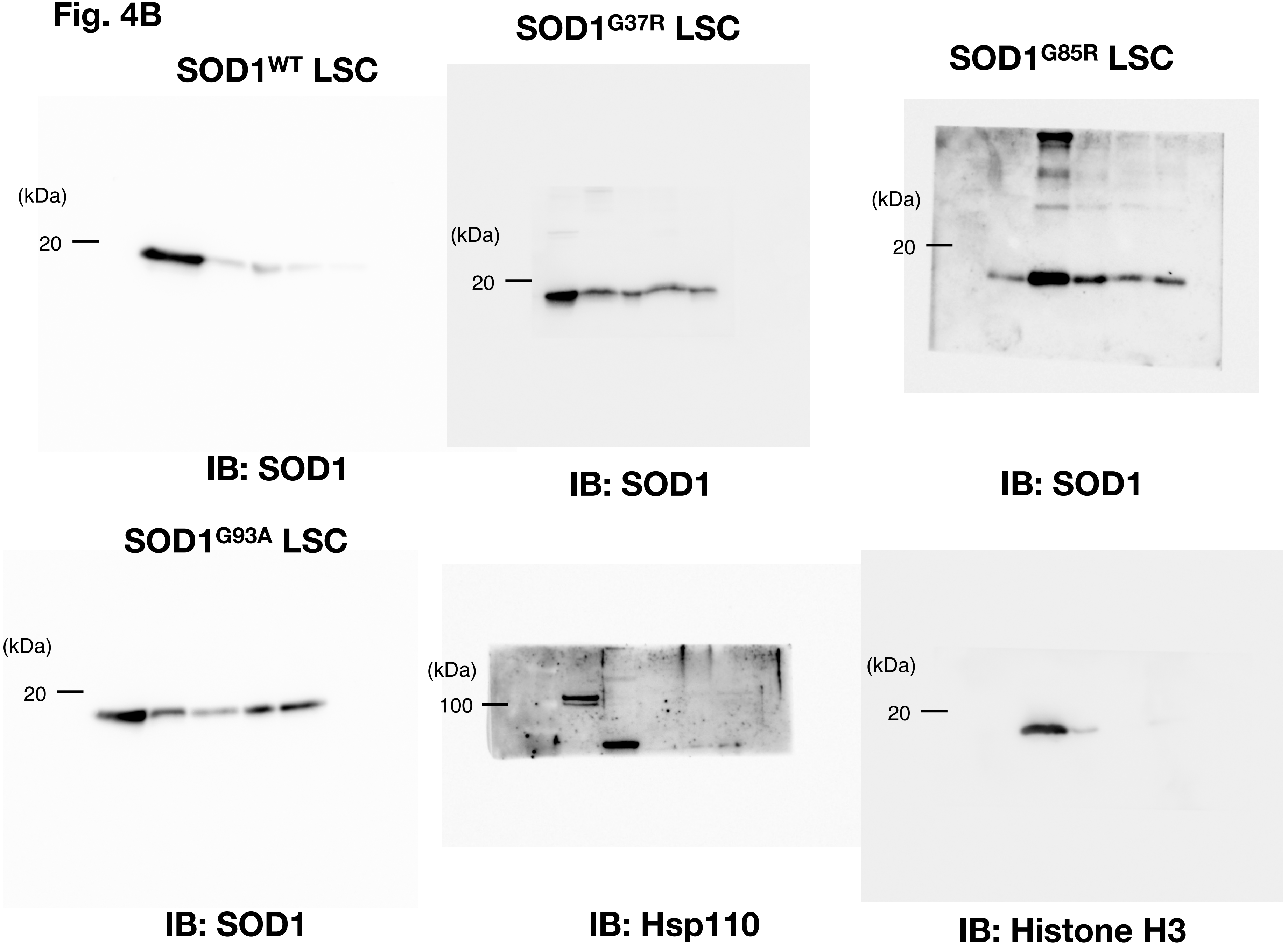
SOD1^{WT} LSC
(kDa)
20
IB: SOD1
SOD1^{G37R} LSC
(kDa)
20
IB: SOD1
SOD1^{G85R} LSC
(kDa)
20
IB: SOD1
SOD1^{G93A} LSC
(kDa)
20
IB: SOD1
(kDa)
100
IB: Hsp110
(kDa)
20
IB: Histone H3

Fig. 4B

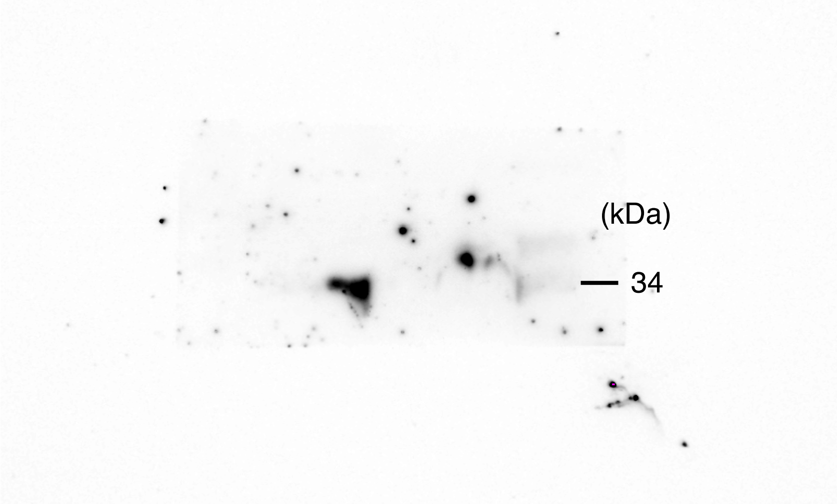

IB: VDAC

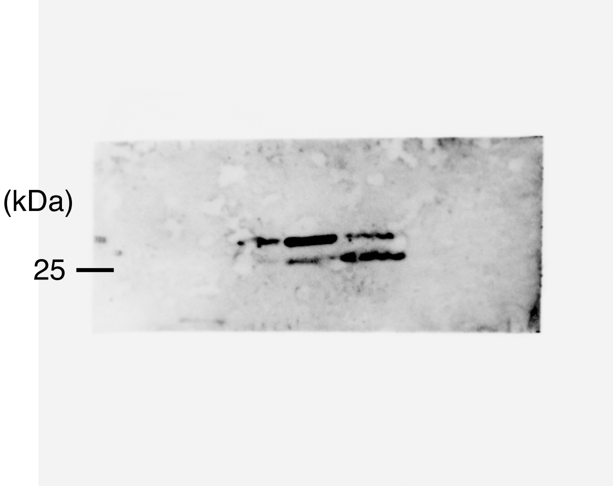

IB: Sig1R

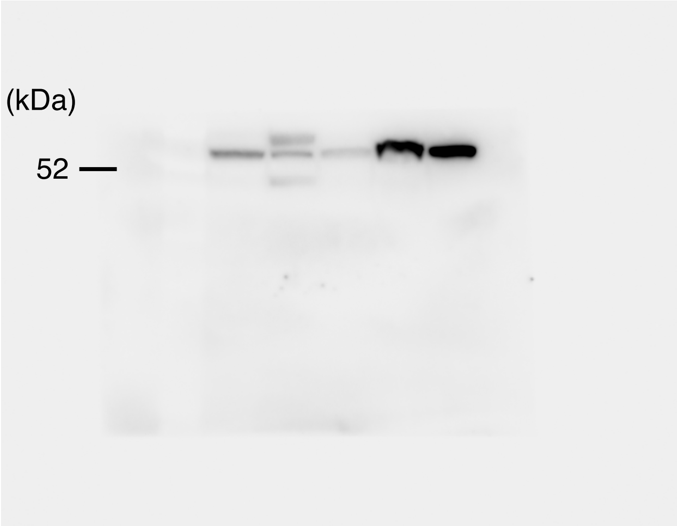

IB: PDI

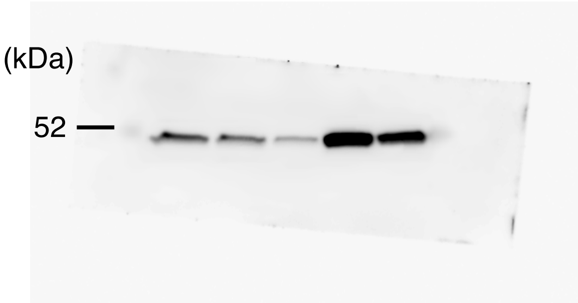

IB: Calreticulin

Fig. 4B

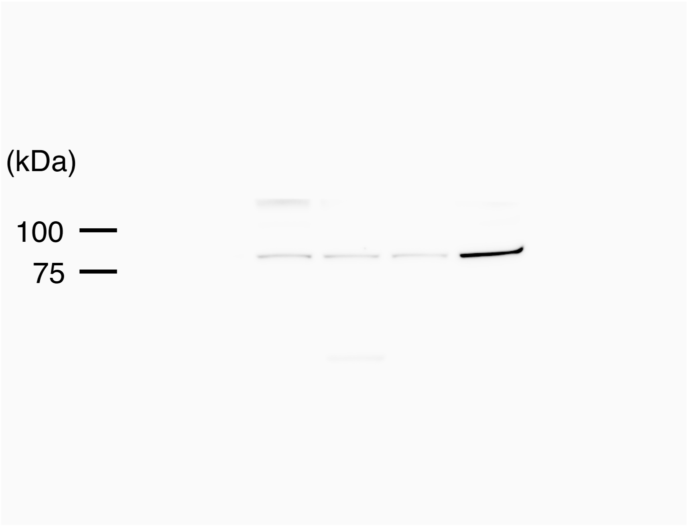

IB: CYPOR

Fig. 4C

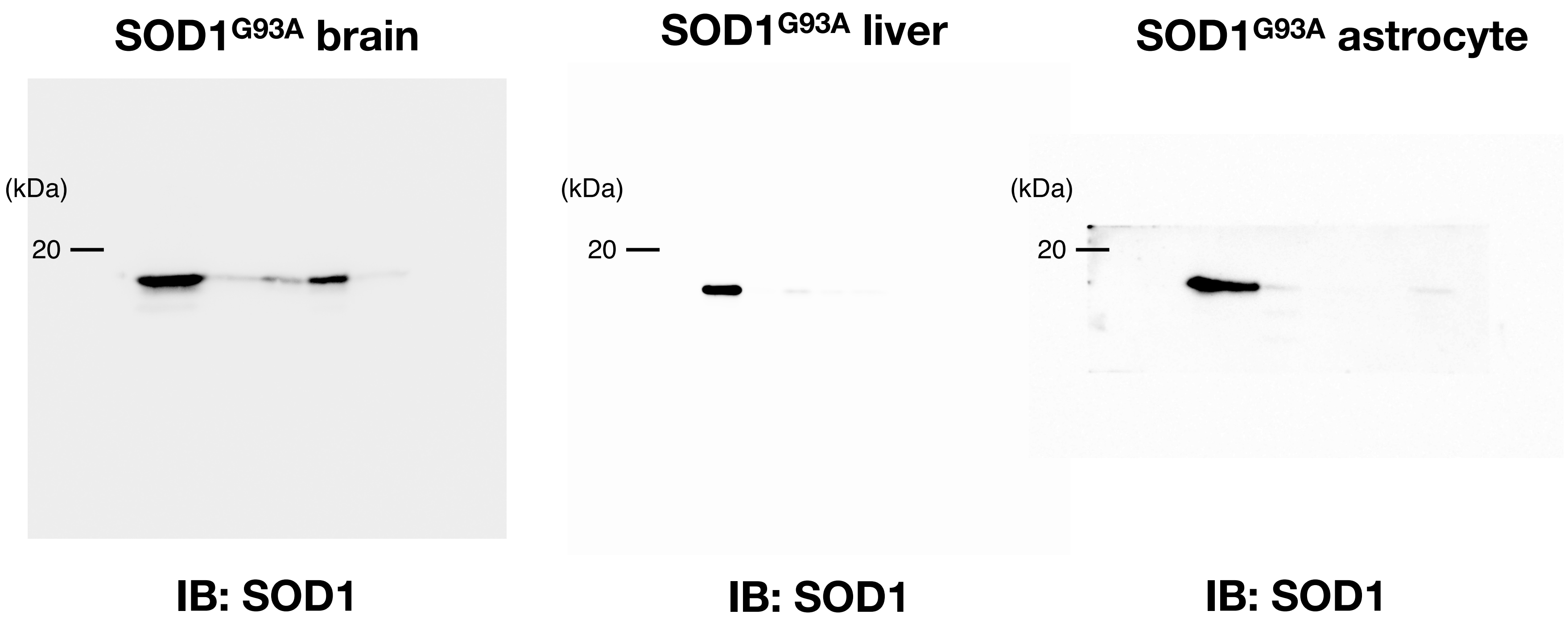
SOD1G93A brain
SOD1G93A liver
SOD1G93A astrocyte
(kDa)
20
(kDa)
20
(kDa)
20
IB: SOD1
IB: SOD1
IB: SOD1

Fig. 4D

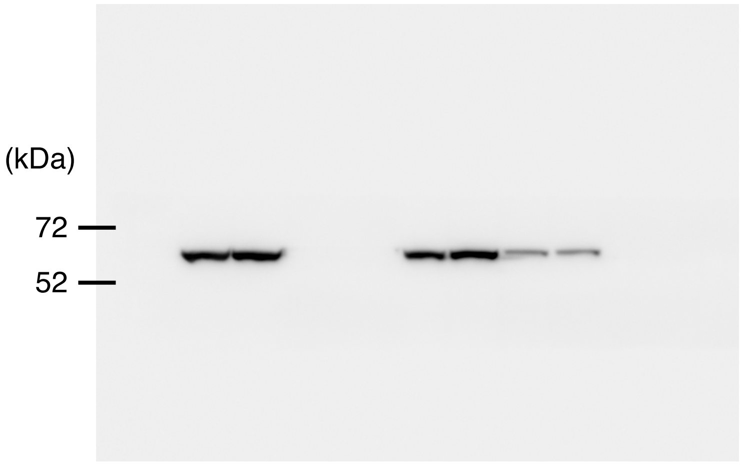

IB: PDI

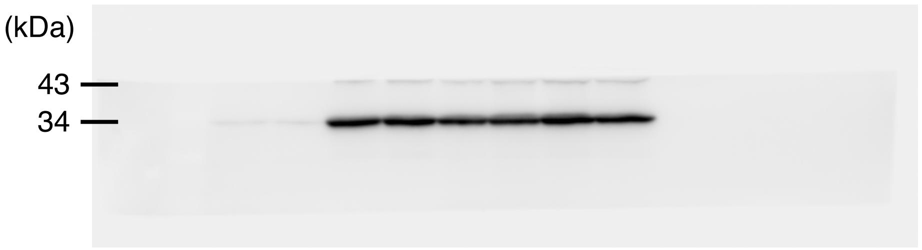

IB: VDAC
