# Supplementary material for: Mitochondria‐associated membrane collapse is a common pathomechanism in SIGMAR1‐ and SOD1‐linked ALS
Source: EMBO Mol Med. 2016 Nov 7;8(12):1421–37. doi: 10.15252/emmm.201606403 (PMC5167132; doi:10.15252/emmm.201606403)
Supplement: Supplementary file 8 — Source Data for Figure 5 [file EMMM-8-1421-s006.pdf]

Fig. 5A

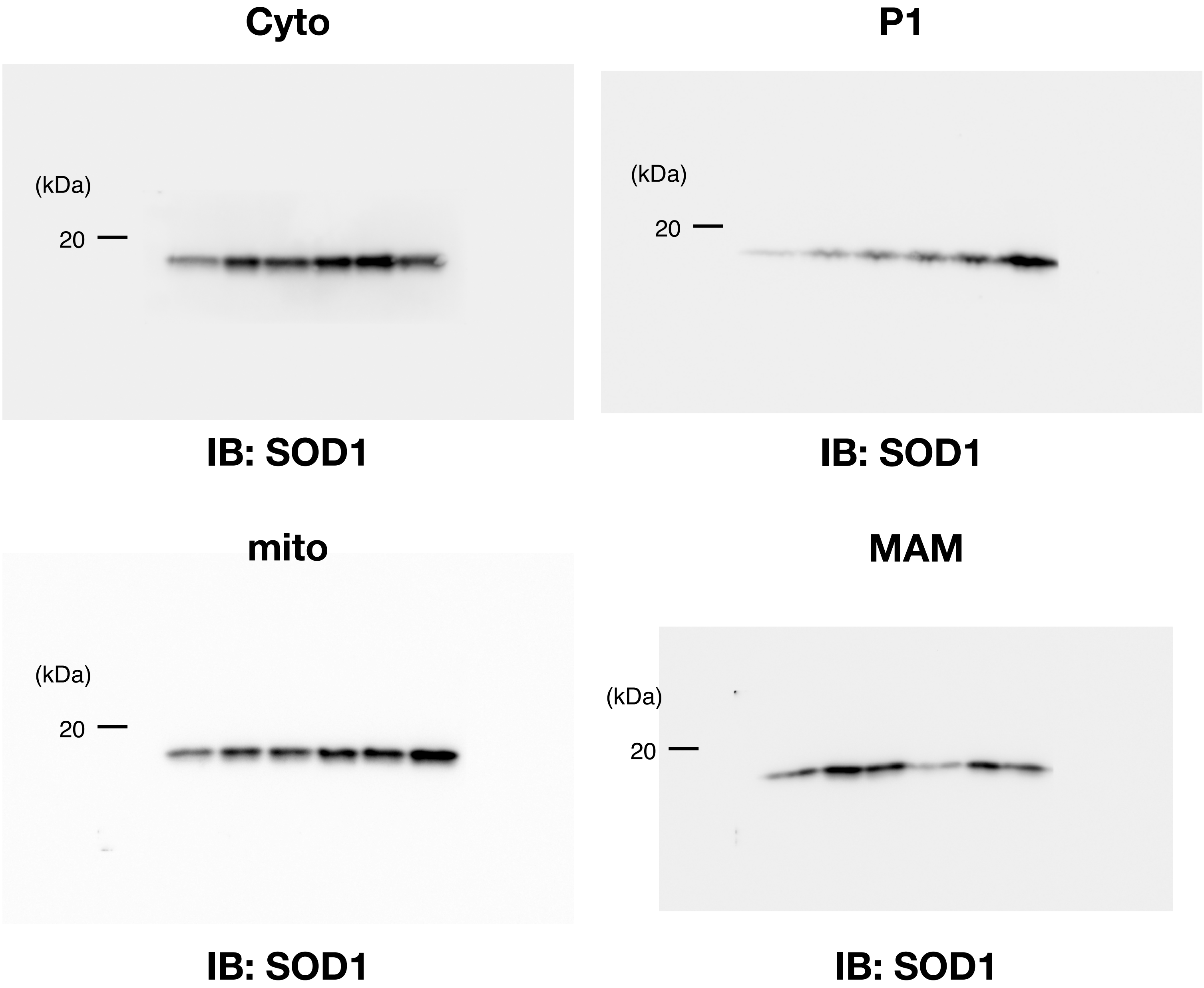
Cyto
(kDa)
20
IB: SOD1
P1
(kDa)
20
IB: SOD1
mito
(kDa)
20
IB: SOD1
MAM
(kDa)
20
IB: SOD1

Fig. 5A

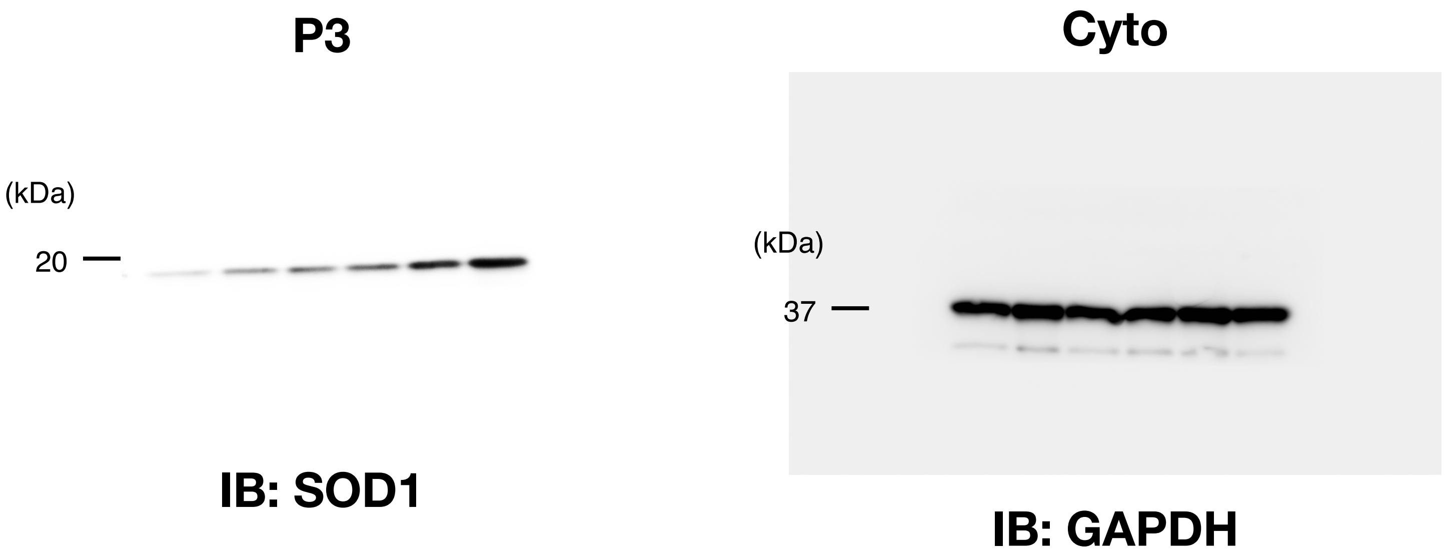

Fig. 5B

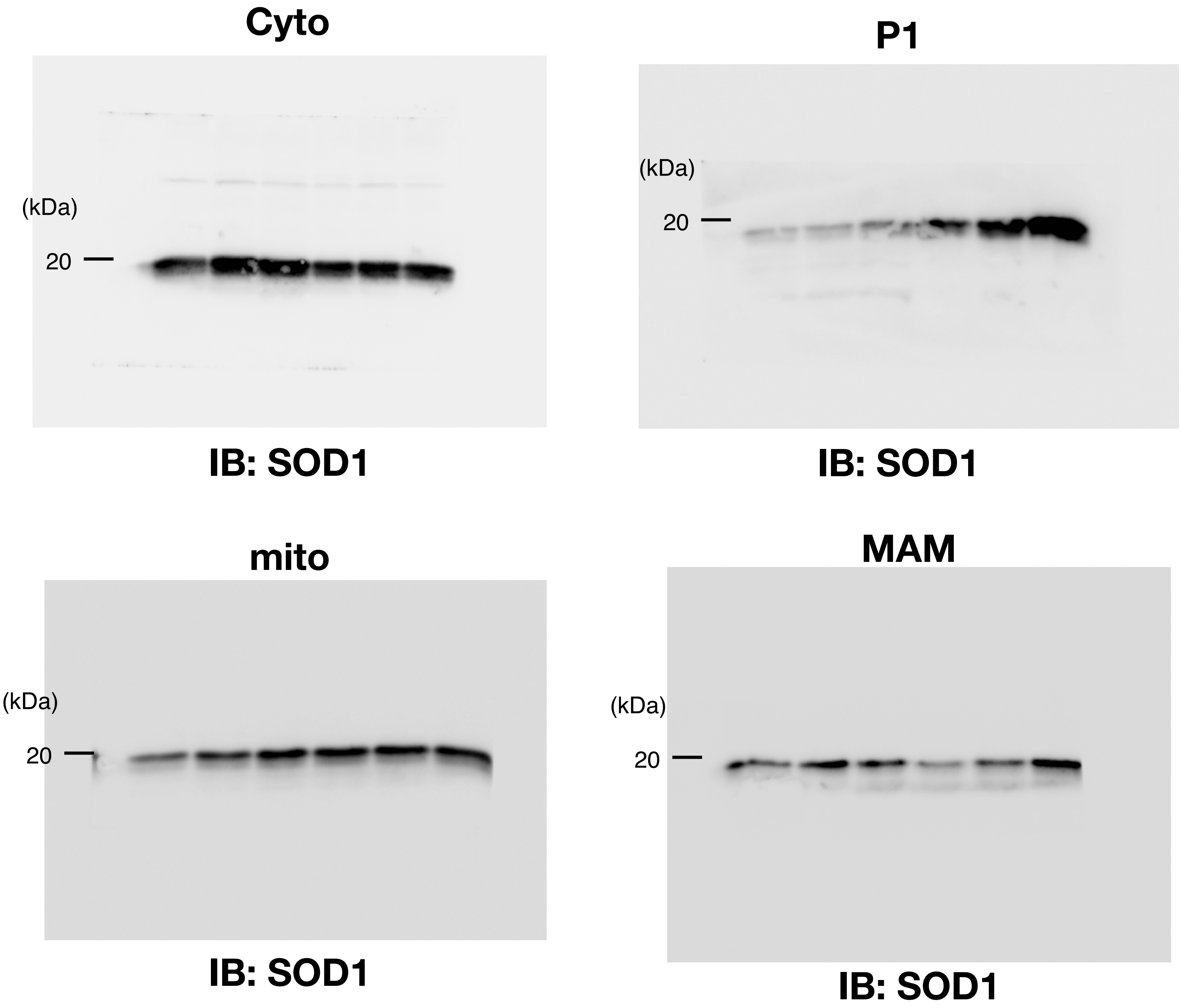
Cyto
(kDa)
20
IB: SOD1
P1
(kDa)
20
IB: SOD1
mito
(kDa)
20
IB: SOD1
MAM
(kDa)
20
IB: SOD1

Fig. 5B

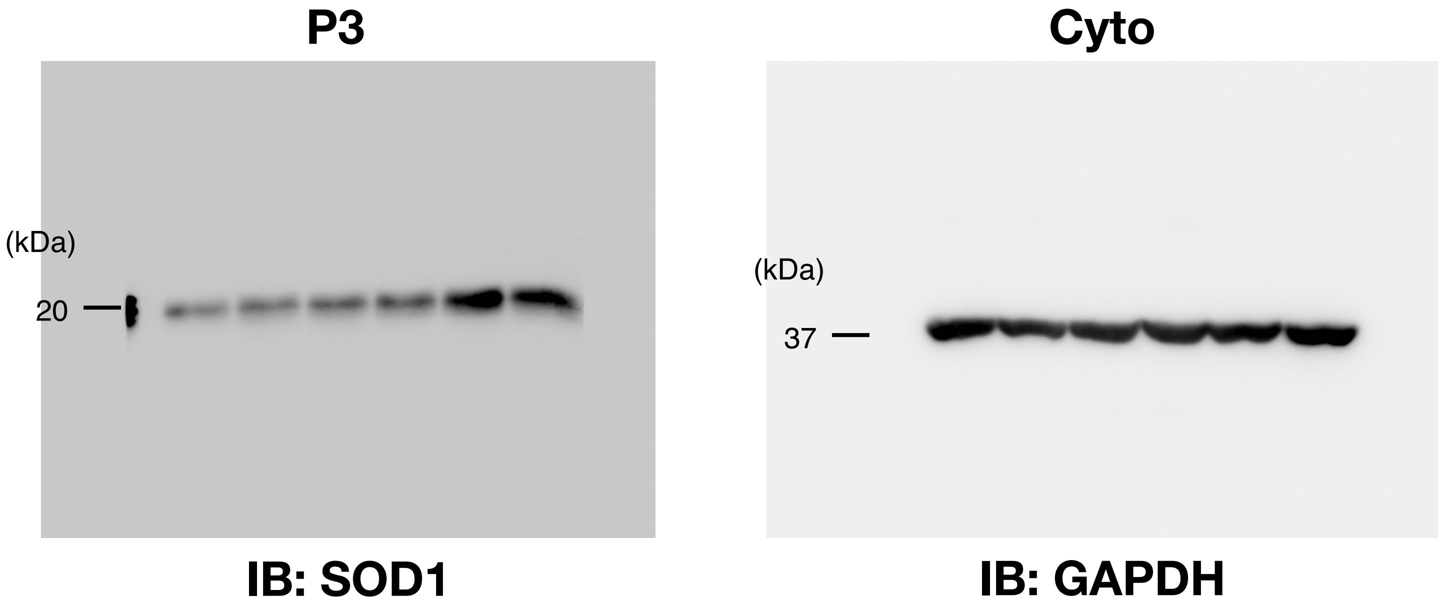

Fig. 5C

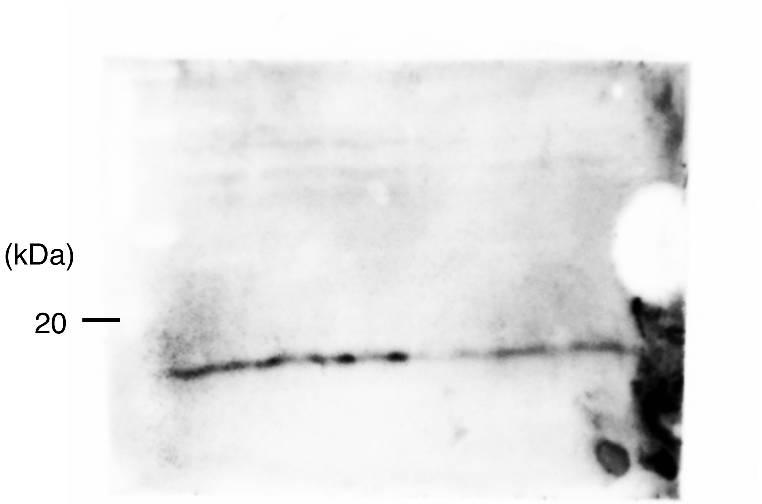

IB: hSOD1

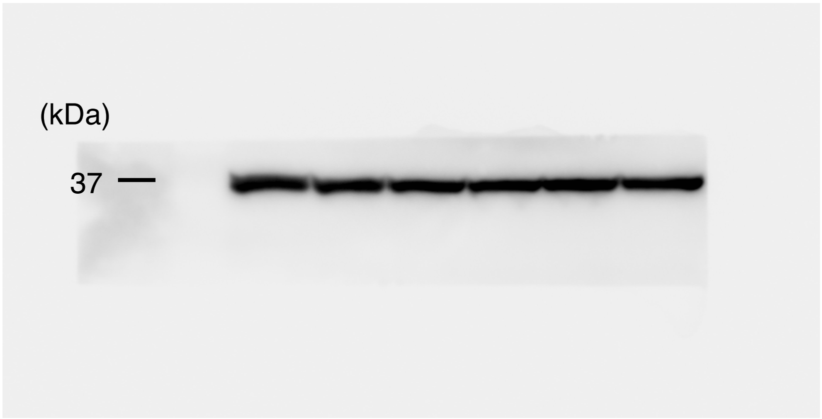

IB: GAPDH

Fig. 5D

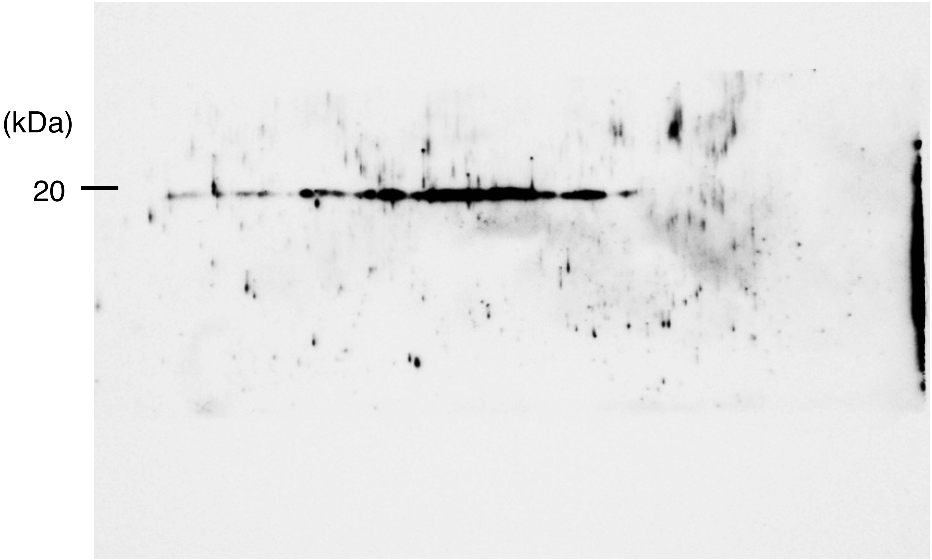

IB: hSOD1

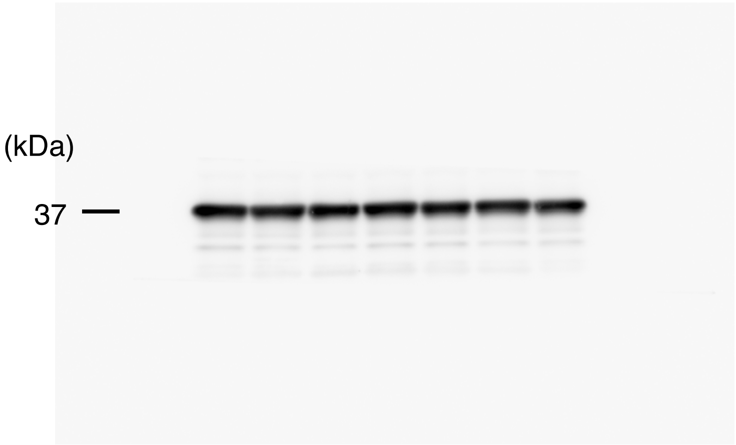

IB: GAPDH

Fig. 5E

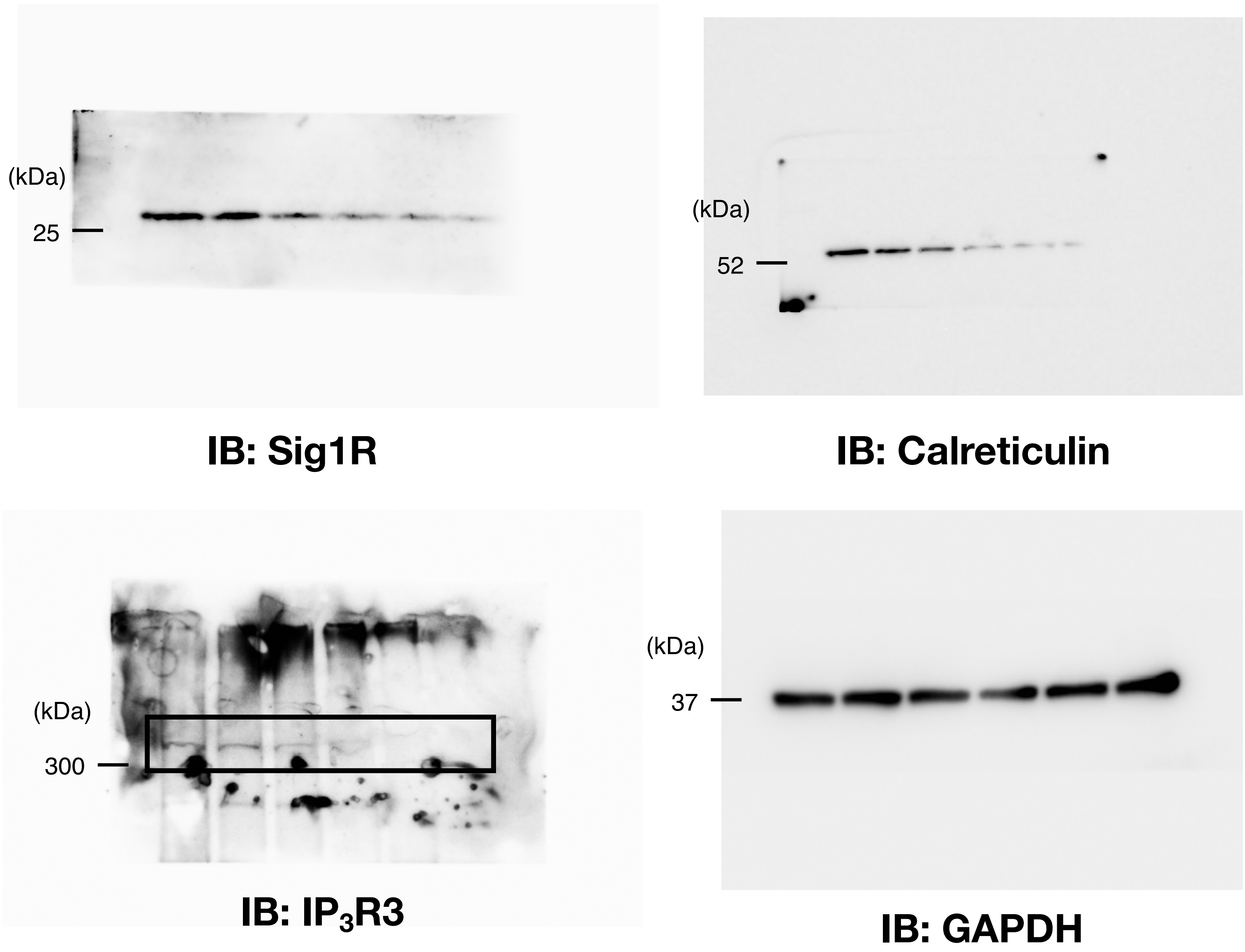
(kDa)
25
IB: Sig1R
(kDa)
52
IB: Calreticulin
(kDa)
300
IB: IP3R3
(kDa)
37
IB: GAPDH

Fig. 5E

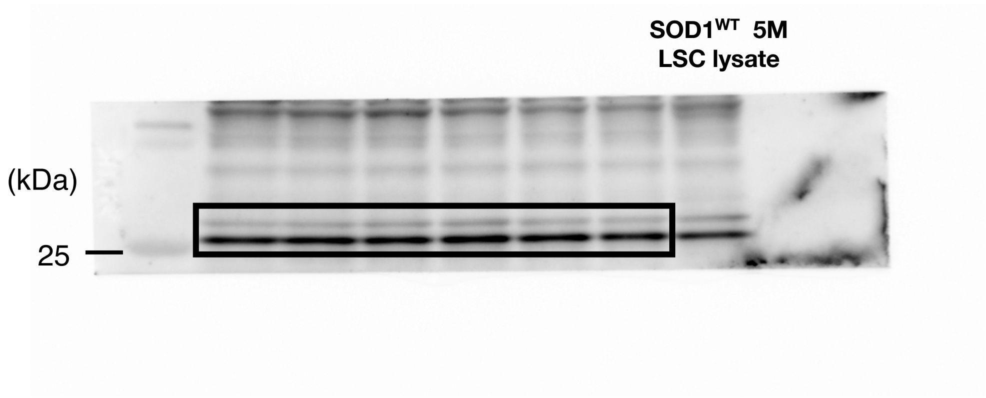

IB: Sig1R

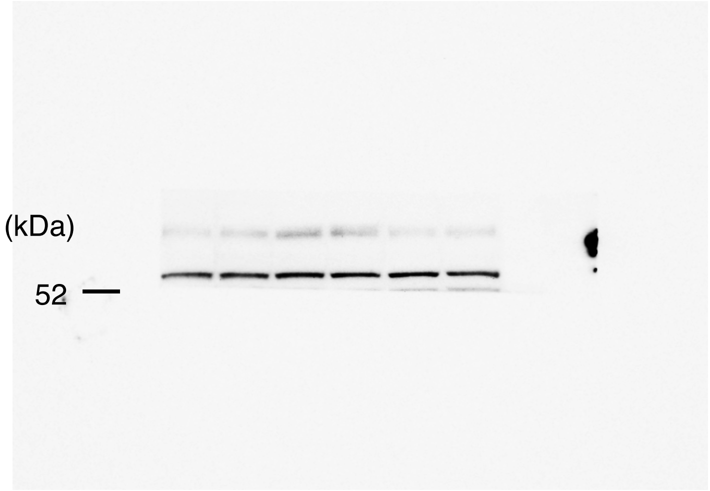

IB: Calreticulin

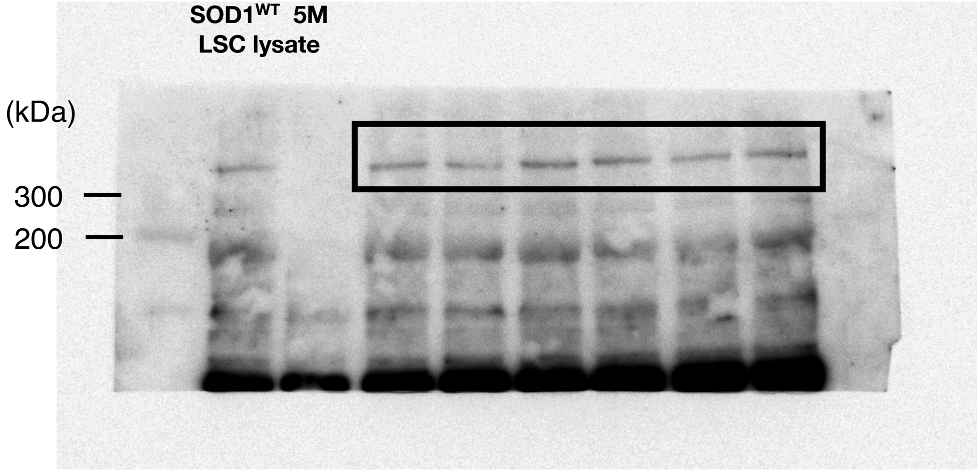

IB: IP_3R3

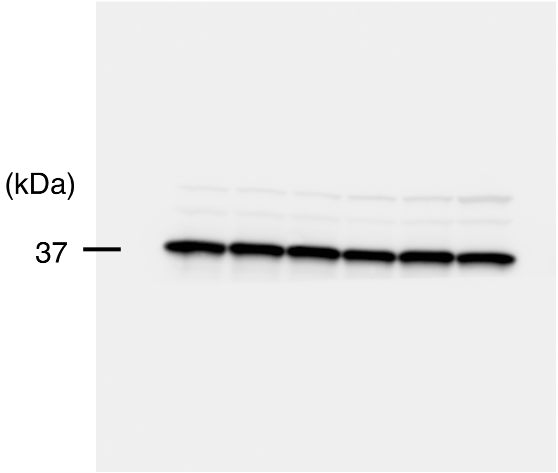

IB: GAPDH
